# Narrowing the Gap Between *In Vitro* and *In Vivo* Genetic Profiles by Deconvoluting Toxicogenomic Data *In Silico*

**DOI:** 10.3389/fphar.2019.01489

**Published:** 2020-01-08

**Authors:** Yuan Liu, Runyu Jing, Zhining Wen, Menglong Li

**Affiliations:** ^1^ College of Chemistry, Sichuan University, Chengdu, China; ^2^ College of Cybersecurity, Sichuan University, Chengdu, China

**Keywords:** toxicogenomics, nonnegative matrix factorization, *in vitro* to *in vivo* extrapolation, *in vivo* and *in vitro* strategies, gene expression, deconvolution, liver, bioinformatics

## Abstract

Toxicogenomics (TGx) is a powerful method to evaluate toxicity and is widely used in both *in vivo* and *in vitro* assays. For *in vivo* TGx, reduction, refinement, and replacement represent the unremitting pursuit of live-animal tests, but *in vitro* assays, as alternatives, usually demonstrate poor correlation with real *in vivo* assays. In living subjects, in addition to drug effects, inner-environmental reactions also affect genetic variation, and these two factors are further jointly reflected in gene abundance. Thus, finding a strategy to factorize inner-environmental factor from *in vivo* assays based on gene expression levels and to further utilize *in vitro* data to better simulate *in vivo* data is needed. We proposed a strategy based on post‐modified non‐negative matrix factorization, which can estimate the gene expression profiles and contents of major factors in samples. The applicability of the strategy was first verified, and the strategy was then utilized to simulate *in vivo* data by correcting *in vitro* data. The similarities between real *in vivo* data and simulated data (single-dose 0.72, repeat-doses 0.75) were higher than those observed when directly comparing real *in vivo* data with *in vitro* data (single-dose 0.56, repeat-doses 0.70). Moreover, by keeping environment-related factor, a simulation can always be generated by using *in vitro* data to provide potential substitutions for *in vivo* TGx and to reduce the launch of live-animal tests.

## Introduction

*In vivo* and *in vitro* experimental systems are two essential ways to discover functional performance during drug-discovery, vital processes occurring in living organisms and toxicological research (Nuwaysir et al., 1999). For animal trials, *in vivo* experiments still can be launched properly for toxicological research (Hussain et al., 1987; Liu et al., 2017). The only problem is that *in vivo* costs are higher than those for *in vitro* assays, so it is more difficult to utilize *in vivo* systems in large-scale projects. However, for human trials, *in vivo* studies can only be applied in the field if they cause no damage to the human body, such as the studies in the brain science (Plate et al., 1992; Glasser and Van Essen, 2011; Zhang et al., 2012), neuroscience (Fagan et al., 2006; Swain et al., 2007), and cell behavior observation fields (Gronthos et al., 2000; Pillay et al., 2010; Eskildsen et al., 2011). More specifically, in toxicology studies, *in vivo* assays are currently difficult to apply in human trials because of the high risk to human volunteers or patients.

With the recent rapid development of genomics, toxicogenomics (TGx), which is a combination of toxicology and genomics technologies, has become a powerful method to study the underlying molecular mechanisms of toxicity (Aardema and MacGregor, 2003). TGx gives a novel perspective to investigate and predict toxicology (Nuwaysir et al., 1999; Waters and Fostel, 2004), risk assessment (Pennie et al., 2004; Boverhof and Zacharewski, 2005), and mechanistic studies (Suter et al., 2004; Suter et al., 2011; Chen et al., 2012a). Moreover, with the development of emerging technologies, novel genomic TGx features, such as microRNAs (Wang et al., 2009; Yang et al., 2011) and lncRNAs (Aigner et al., 2016; Dempsey and Cui, 2017), may provide a new way to achieve more resolution and better understand the mechanisms of toxicology. However, the fact that *in vivo* assays have high costs and are difficult to apply in humans still persists with TGx and has hindered the development of toxicology.

The ideal situation is that an *in vivo* system can be supported or even reflected by a low-cost approach or experiment involving animal reduction (Iwatsubo et al., 1997; Rostami-Hodjegan and Tucker, 2007). To reach this goal, decades of studies have tried approaches using different data and models. Among all the approaches, obtaining models from *in vitro* cells or tissue cultures (IVIVE—*in vitro* to *in vivo* extrapolation) has become a main alternative (Knight et al., 2006). For utilizing cells and tissue cultures, the concept of “the 3Rs of alternatives” (reduction, refinement, and replacement) (Russell et al., 1959) was first described. Based on the 3Rs, worldwide organizations are trying to develop methods to achieve IVIVE by using TGx. These methods include the Registration, Evaluation, Authorization and Restriction of Chemicals (REACH) program launched in Europe (Abbott, 2005), “Advancing Regulatory Science” initiated by the Food and Drug Administration (FDA) (Hamburg, 2011) of the United States, and a guideline for the use of the single‐cell gel (SCG)/Comet assay developed by the International Workshop on Genotoxicity Test Procedures (IWGTP) to standardize genetic toxicology procedures (Tice et al., 2000). Additionally, other relevant programs have been launched by organizations, such as ICH (International Conference on Harmonization of technical requirements for the registration of pharmaceuticals for human use), CPCSEA (Committee for the Purpose of Control and Supervision of Experiments on Animals), NIH (National Institutes of Health), and OECD (Organization for Economic Co-operation and Development) (Rollin, 2003; Abbott, 2005; Dix et al., 2007; Hamburg, 2011; Tice et al., 2013).

With worldwide contributions, the capability of *in vitro* data for providing assistance and references for *in vivo* data has approved substantially. For instance, IVIVE can be achieved by using physiologically-based pharmacokinetic (PBPK) modeling (Chen et al., 2012b; Sager et al., 2015) on TGx (Alam et al., 2013; Martin et al., 2014). Additionally, human health risk can be assessed based on TGx analysis (Johnson et al., 2015). A demonstration showed that liver sections exhibit the strongest similarity to liver tissue in terms of mRNA expression (Boess et al., 2003). Reviews confirmed that TGx is able to improve comprehension of the mechanisms underlying the responses of *in vitro* and *in vivo* systems (Poma and Di Giorgio, 2008). Moreover, *in vitro* data predicted carcinogenesis in rats based on short-term TGx data (Watanabe et al., 2007; Ellinger-Ziegelbauer et al., 2008). To summarize, *in vitro* assays as alternatives for TGx play a vital role in the next-generation risk assessment paradigm and have tremendous potential to promote non-animal testing in TGx systems (Liu et al., 2018c).

However, the goal of using *in vitro* data to substitute for *in vivo* data is greatly impeded by the inconsistency between *in vivo* and *in vitro* data (Niki, 2010). Many researchers are challenged by the fact that *in vitro* data demonstrate poor correlations with *in vivo* data and have questioned the validity of IVIVE models (Tice et al., 2000; Chen et al., 2014; Sutherland et al., 2016). Previous research has stressed that many available methods result in inconsistent results regarding antioxidant capacity between *in vivo* and *in vitro* data (Niki, 2010). Laboratory technicians have even mentioned that the *in vivo* dynamics of antigen-specific regulatory T cells cannot be predicted from *in vitro* behavior (Klein et al., 2003). Some publications showed that the data obtained from *in vivo* livers without the use of chloroquine was inconsistent with *in vitro* gene expression results when using cultured HepG2 cells (Harada-Shiba et al., 2002). After assessing the capacity of *in vitro* screening studies to predict the *in vivo* pulmonary toxicity of several fine or nanoscale particles in rats, a poor correlation between *in vivo* and *in vitro* studies was observed (Sayes et al., 2007). The pair ranking (PRank) method (Liu et al., 2017) was proposed by the National Center for Toxicological Research of the U.S. FDA to assess IVIVE and to quantitatively measure similarity (Chen et al., 2012a; Chen et al., 2014). The PRank method has made it possible to properly compare gene expression data between *in vivo* and *in vitro* assays. However, the similarities between *in vitro* and *in vivo* data are unsatisfactory. The similarities between *in vivo* and *in vitro* data still can be further improved, especially for *in vivo* single-dose studies.

According to current investigations, in addition to the response signals from drug effects, many variables need to be considered inner-environmental factors that impact genetic variations in *in vivo* assays, such as cell types, culture conditions, time course of exposure, and measured end points (Sayes et al., 2007). Differences are found when demonstrating the immune response components (effectors) of *in vivo* and *in vitro* hepatocytes (Bumgardner et al., 1990). It has been indicated that the inner environment of a living subject has different patterns of physiological function and mechanism. Additionally, therapy–pharmacokinetics (Harada-Shiba et al., 2002), pharmacokinetics and pharmacodynamics (PK/PD) (Liu et al., 2017) are usually not considered within the proposed IVIVE assessment of toxicogenomic data. Eventually, with the comprehensive effects and variables that are determined by the complicated inner-environment of *in vivo* data, an inconsistent gap has emerged between *in vivo* data and *in vitro* data, and this inconsistency is further reflected at the level of gene expression. Thus, based on TGx data, the properties of a molecule can influence a drug effect that ultimately reaches the cell in different assays (Groothuis et al., 2015; Kramer et al., 2015). Furthermore, the differences in the response components of a living system are reflected differently between gene expression profiles from *in vivo* and *in vitro* data.

To achieve better utilization of IVIVE, it is urgently necessary to find a strategy that can extract the inner-environment factor from *in vivo* TGx data, and based on that, to further develop a valid strategy that is able to more accurately simulate *in vivo* data from *in vitro* data. Non‐negative matrix factorization (NMF) (Lee and Seung, 1999; Lee and Seung, 2001), consisting of a series of unsupervised learning methods, is a classical method to factorize a matrix to nonnegative matrixes. NMF is commonly utilized to reduce the computational consumption or dimensions of data, and to filter specific markers or gene selection for genetic data (Dai et al., 2006). As for *in vivo* and *in vitro* data, NMF has been utilized to image analysis, feature selection, and cancer-type classify (Maruyama et al., 2014; Shourijeh et al., 2016). In a previous study, we proposed a post-modified NMF approach to make NMF more suitable for the utilization of biological systems and further applied this approach for deconvoluting gene expression profiles of cancer samples (Liu et al., 2018a). Post-modified NMF is capable of estimating the gene expression profiles and contents of the major factors in samples without any prior reference knowledge. Therefore, post-modified NMF has the potential to factorize major factors based on *in vivo* gene expression profiles.

In this study, we developed a strategy that can extract the inner-environment factor from *in vivo* TGx data at the gene expression level. We first verified the applicable of Post-modified NMF on *in vivo* and *in vitro* profiles. The strategy is able to factorize *in vivo* data into “drug-responding component” and “environmental component” by using post-modified NMF. And then, by combining *in vitro* data with the “environmental component” factorized from *in vivo* data, simulated *in vivo* data was obtained. The results indicated that the simulated *in vivo* data is more compatible with the original *in vivo* data than the use of *in vitro* data directly, and can be utilized to narrow the gap between *in vivo* and *in vitro* data on the gene expression level.

## Results

### Study Design

We sought to verify the capability of the deconvolution method to decrease the inconsistency between *in vivo* and *in vitro* TGx data and to develop a valid simulation for *in vivo* data using *in vitro* data. All investigations involved two stages: verification and simulation. The verification stage entailed evaluating whether post-modified NMF can be utilized to factorize the inner-environmental factors of *in vivo* data from TGx data. The simulation stage narrowed the gap between two assay types, so that *in vivo* data can be simulated better by *in vitro* assays.

We collected *in vivo* data (single dose/repeat doses) and *in vitro* data (**Table 1**) on 170 compounds from The Open Japanese Toxicogenomics Project-Genomics Assisted Toxicity Evaluation System (Open TG-GATEs) (Uehara et al., 2010; Igarashi et al., 2014). In the verification stage, two group comparisons based on three systems were carried out: *in vivo* single VS *in vitro* and *in vivo* repeat VS *in vitro*. In the simulation stage, two group comparisons based on *in vivo* systems were carried out: *in vivo* single VS original *in vivo* and *in vivo* repeat VS original *in vivo*.

**Table 1:** Data information and usage in this study.

**Species—platform**	Assay	System	Tissues	Number of compounds	Time points	Dose^*^	Number of main factors	Involved data	
								Original	Deconvoluted
**Sprague-Dawley rat (6 weeks old)—Affymetrix GeneChip^®^ Rat Genome 230 2.0 Array**	*In vitro*	*In vitro*	Primary hepatocytes	144	2, 8, 24 h	Control, low, middle, high (0:1:5:25)	1	*In vitro*	None
	*In vivo*	*In vivo* (single dose)	Liver	158	3, 6, 9, 24 h	Control, low, middle, high (mainly 0:1:3:10)	2	Original *In vivo* (single dose)	Drug-responding component
									Environmental component
		*In vivo* (repeat doses)	Liver	143	24 h after the last dose after repeated treatment for 3, 7, 14, 28 days	Control, low, middle, high (mainly 0:1:3:10)		Original *in vivo* (repeat doses)	Drug-responding component
									Environmental component

^*^For the in vitro assay, the highest concentration was defined as the dose level generating an 80–90% relative survival ratio with a ratio of 1:5:25 for the low, middle, and high concentration levels. For the in vivo assay, the highest dose was chosen to match the level that induces the minimum toxic effect in a 4-week toxicity study. Then, the ratio of the low, middle, and high dose levels was appropriately set as 1:3:10.

**Figure 1:**
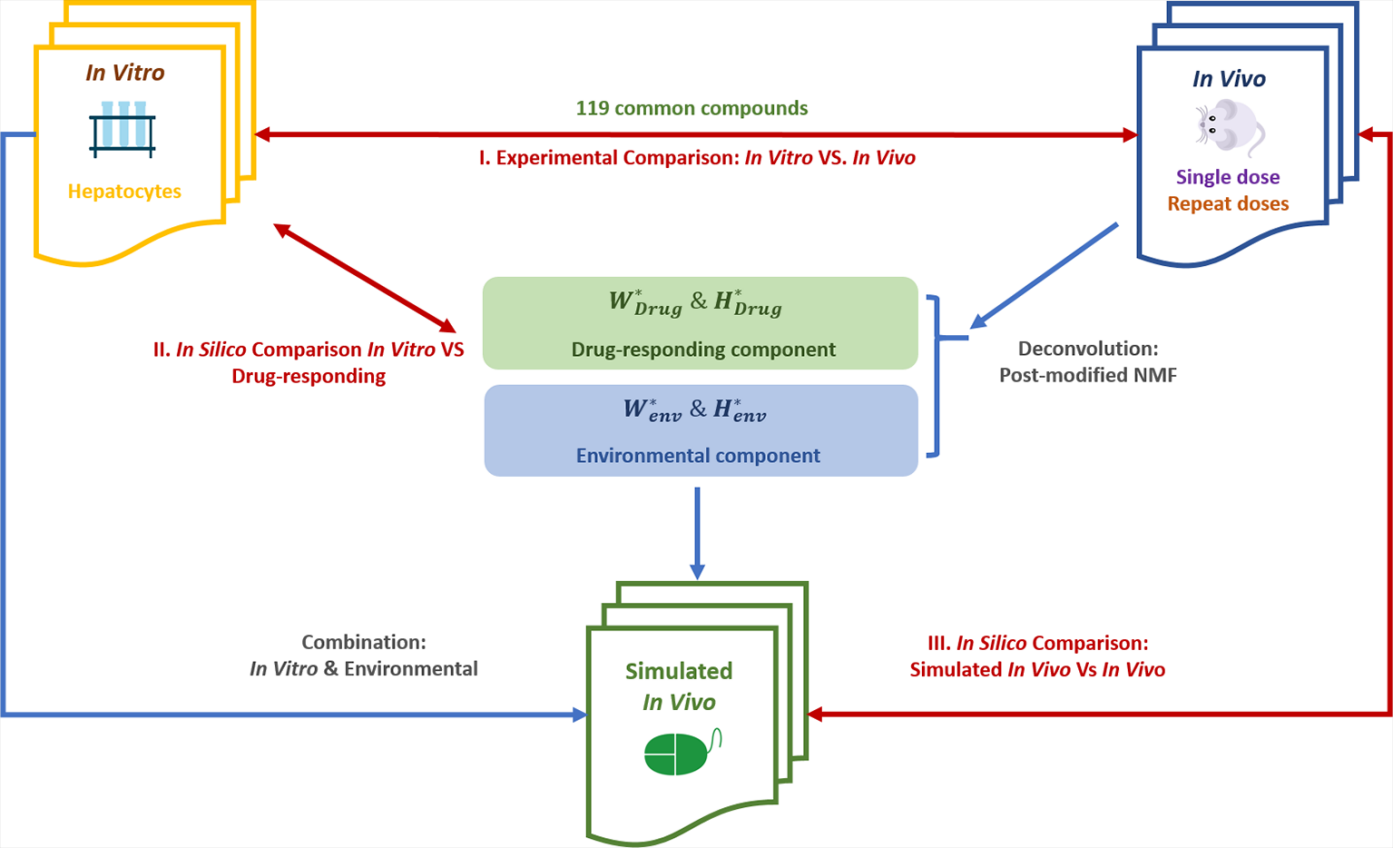
The flowchart of our study design. Post-modified NMF, post‐modified non‐negative matrix factorization; comparisons accomplished by using the pair ranking method (PRank score) and other indicators. $W_{drug}^{*}$, $H_{drug}^{*}$, $W_{env}^{*}$, $H_{env}^{*}$ were deconvoluted from experimental *in vivo* data.

**Figure 1** shows the overall flowchart of our study, including the data and analytical approaches for both the verification stage and application stage.

### *In Vivo* “Drug-Responding Component” Showed Higher Similarity With *In Vitro* Data

In the verification stage, two group comparisons based on three systems were carried out: *in vivo* single dose VS *in vitro* and *in vivo* repeat doses VS *in vitro*. The deconvolution approach was launched to factorize and verify two main factors of the *in vivo* data. Two factors related to body-environment and drug-response in *in vivo* systems were named “environmental component” and “drug-responding component,” respectively.

Using factor analysis, our study first confirmed that the expression data of both *in vivo* systems (single dose and repeat doses) contained two main factors (**Figure S1**), whereas the *in vitro* system only had one main factor (discussed in *Confirm the Number of Factors (k Value) for Deconvoluting by Factor Analysis*). When deconvoluting *in vivo* data by post-modified NMF, the matrix of the gene expression profiles of each compound was factorized into two matrices: W* represented the gene expression profiles, and H* represented the weights of the two main factors. Additionally, for the two lines in W*, the line that had a higher correlation (Pearson’s correlation coefficient) with corresponding control profiles was profiled as “environmental component,” and the other line that had less correlation with corresponding control profiles was profiled as “drug-responding component.”

**Figure 2:**
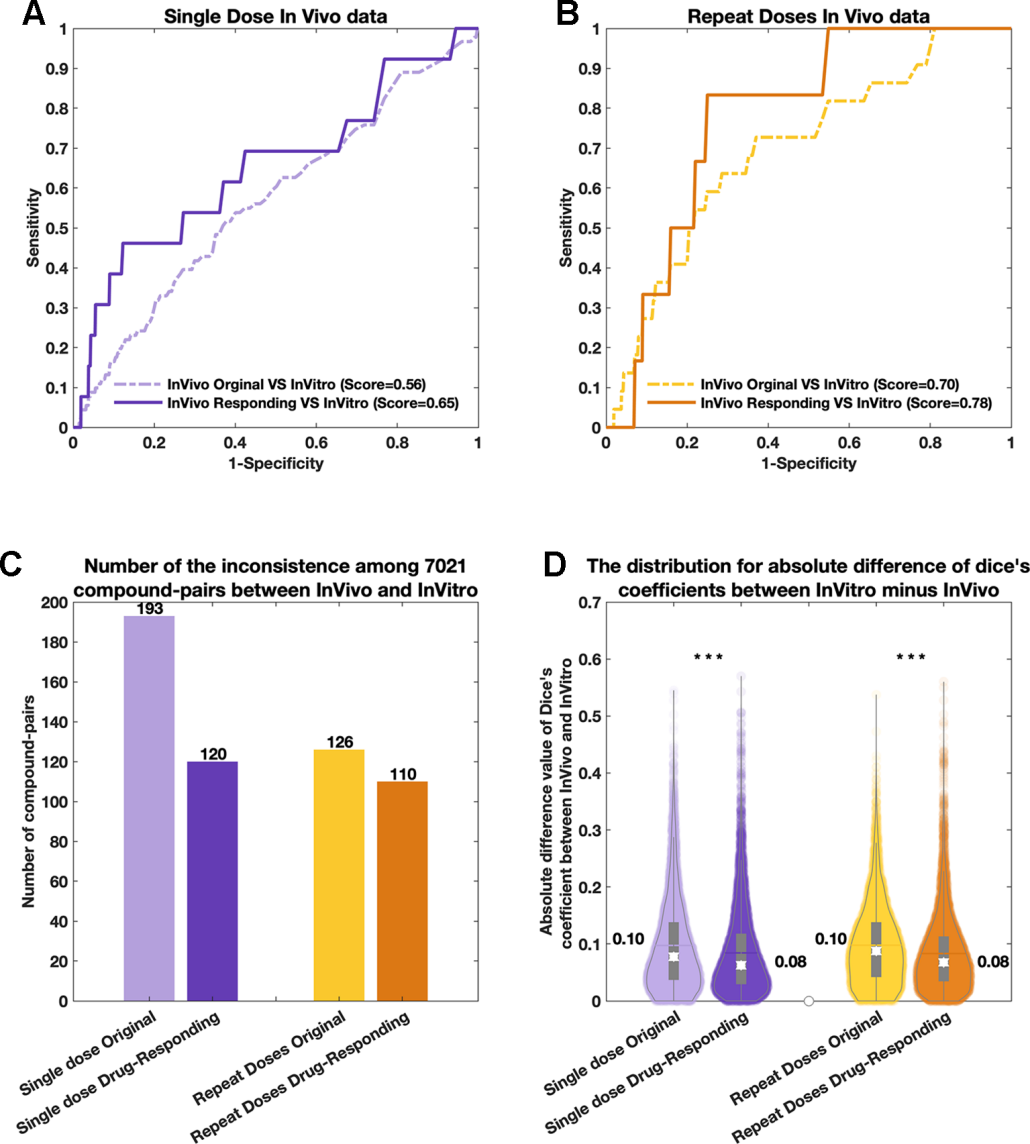
The comparisons among original *in vivo* data, *in vitro* data, and the drug-responding component from the *in vivo* data. For all four subplots, purple represents the single-dose data, and yellow color represents the repeat-doses data. In the comparison with original *in vivo* data, the drug-responding component achieved higher consistencies based on both **(A)** a single dose and **(B)** repeat doses. **(C)** The comparison of the number of inconsistent compound-pairs between the *in vivo* and *in vitro* data among each system. **(D)** The violin plots of the absolute difference values between *in vivo* and *in vitro* (|Dice’s coefficient of *in vivo* data—Dice’s coefficient of *in vitro* data|) systems.

As shown in **Figure 2**, based on the comparison of the consistency obtained between the original *in vivo* data and the *in vitro* data, the “drug-responding component” and the *in vitro* data achieved higher consistency during the comparison. Both single-dose and repeat-doses *in vivo* data achieved more than 9% extra similarity when using “drug-responding component” instead of using the original *in vivo* data for comparison with the *in vitro* data. In more detail, we describe three indicators to show the improved consistencies achieved by using “drug-responding component.” 1) We first compared the similarities [PRank score (Liu et al., 2017)] between the *in vivo* (original and “drug-responding component”) and *in vitro* data for each pair of compounds by using the PRank method (**Figures 2A**, **B**). 2) Then, we counted the number of Dice’s coefficients of each compound-pair, revealing different tendencies in *in vivo* (original and “drug-responding component”) and *in vivo* data among 7021 compound-pairs. A different tendency means that Dice’s coefficients are both lower than the cut-off or both higher than the cut-off in the *in vivo* and *in vitro* data (**Figure 2C**). 3) Additionally, in order to investigate the differences in detail, for each compound-pair, we utilized the absolute difference values of Dice’s coefficients to determine how close the gap between the *in vivo* and *in vitro* data was narrowed by deconvoluting the data. Based on 7021 compound-pairs for each system, absolute difference values were obtained by subtracting Dice’s coefficients between “drug-responding component” data and *in vitro* data or between the original *in vivo* data and *in vitro* data. Then, the distribution of absolute differences for each system for single-dose and repeat-doses data could be observed and compared, and the mean absolute difference (MAD) of each system could be calculated as well (**Figure 2D**).

For single-dose *in vivo* data, the similarity of “drug-responding component” VS *in vitro* data was 9% higher than the single-dose original *in vivo* data VS *in vitro* data. The PRank score rose from 0.56 before deconvolution to 0.65 after generating the “drug-responding component” (**Figure 2A**). Additionally, an extra 73 compound-pairs were found when comparing the “drug-responding component” with the *in vitro* data. The number of inconsistent compound-pairs was reduced from 193 to 120 (**Figure 2C**, purple). Furthermore, the MAD values were calculated by subtracting Dice’s coefficients. The “drug-responding component” VS *in vitro* comparison decreased by 0.02 (“drug-responding component” VS *in vitro* = 0.10, and original *in vivo* data VS *in vitro* = 0.08) (**Figure 2D**, purple). The *p-value* of the significance test (Cohen, 1992) (Student t-test) for the absolute difference values between the “drug-responding component” VS *in vitro* and the original *in vivo* VS *in vitro* data was less than 10×10^-10^.

Similar results were achieved with repeat-doses *in vivo* data; the score reached 0.79 for the compression of repeat-doses *in vivo* data “drug-responding component” VS *in vitro* data, while the score obtained from repeat-doses *in vivo* data original VS *in vitro* was 0.70, with 9% higher similarity score (**Figure 2B**). Sixteen extra compound-pairs were found when comparing “drug-responding component” with *in vitro* data. The number of inconsistent compound-pairs was reduced from 126 to 110 among 7021 compound-pairs (**Figure 2C**, yellows). Moreover, a lower tendency was observed when comparing the violin plot of absolute differences obtained by subtracting Dice’s coefficients between the *in vitro* and *in vivo* assays. The MAD value was reduced from 0.10 to 0.08, the gap between the *in vivo* and *in vitro* data decreased by 0.02, and the *p-value* was less than 10×10^-10^ (**Figure 2D**, yellows) when comparing the absolute difference value obtained for the “drug-responding component” VS *in vitro* data and the absolute difference value obtained for the original *in vivo* data VS *in vitro* data, respectively.

To summarize the results of this stage, in both single-dose and repeat-doses data, the “drug-responding component” deconvoluted by post-modified NMF from *in vivo* data showed better results in the investigation among the three indicators than the original *in vivo* data when compared with *in vitro* data. The results indicated that post-modified NMF is able to efficiently factorize the inner-environmental factors and can be used to optimize the consistency between *in vivo* data and *in vitro* data at the gene expression level.

### *In Vivo* “Drug-Responding Component” Can Be Replaced by *In Vitro* Data

In the simulation stage, two group comparisons based on the *in vivo* systems were carried out: *in vivo* single VS original *in vivo* and *in vivo* repeat VS original *in vivo*. Unlike the “drug-responding component,” the “environmental component” tended to reflect attributions that are more related to the inner-environment or biological processing of a living body. Thus, the “environmental component” could be regarded as the main factor for examining the differentiation and inconsistency between *in vivo* data and *in vitro* data. When the “environmental component” was confirmed, simulated *in vivo* data were obtained by combining “environmental component” with *in vitro* data. In other words, *in vitro* data were first used to replace (swap) the “drug-responding component” line in W*, and H* was integrated with the new W* to generate the simulated *in vivo* data.

**Figure 3:**
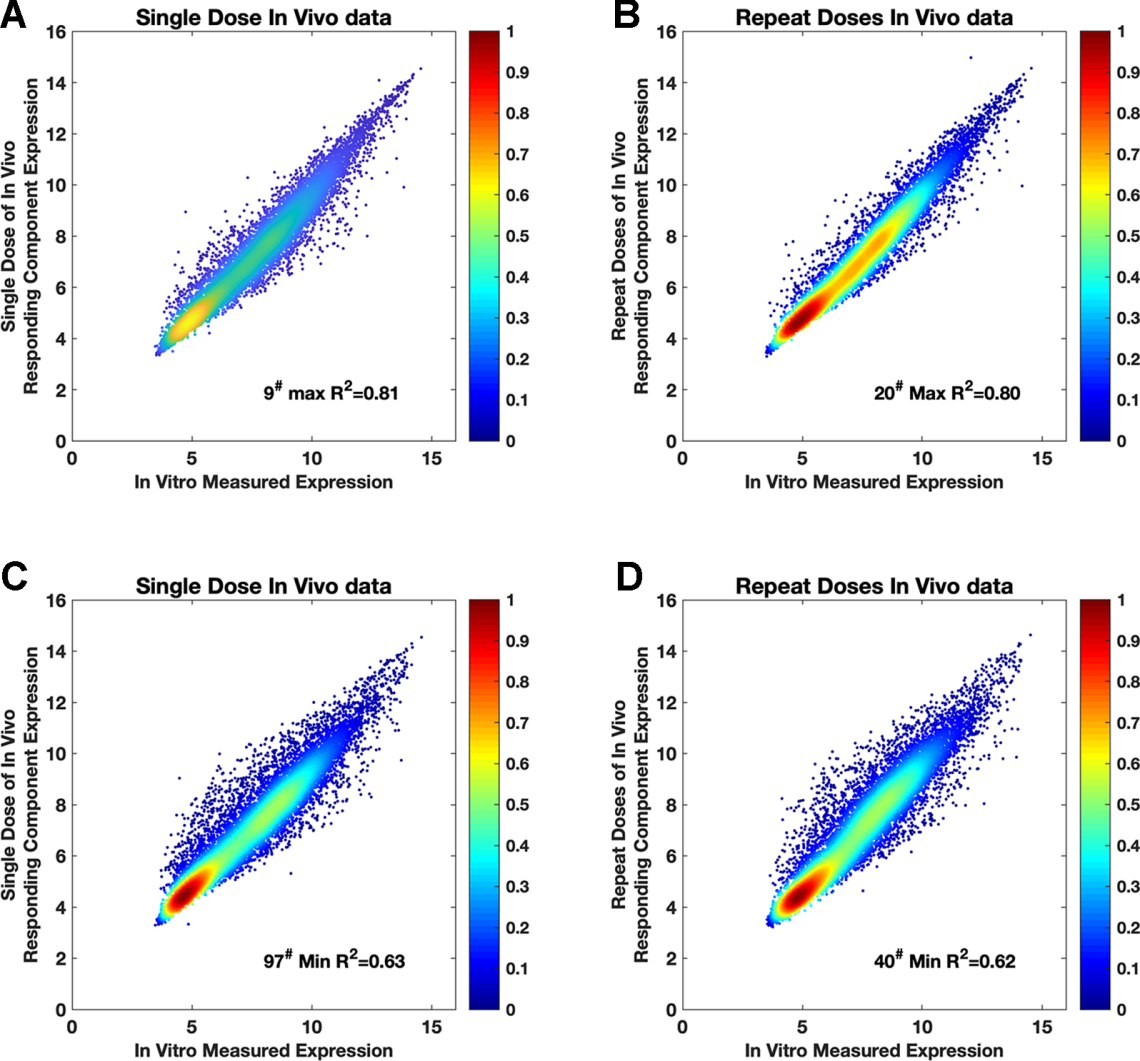
The intensity scatter plots reflecting the consistency between *in vivo* and *in vitro* data. **(A** and **C)** Intensity scatter plots for the compounds that have max R^2^ (R^2^ = 0.81, RMSE = 3.96) and min R2 (R^2^ = 0.63, RMSE = 3.97) in single-dose *in vivo* data. **(B** and **D)** Plots for the compounds that have max R^2^ (R^2^ = 0.80, RMSE = 4.00) and min R2 (R^2^ = 0.62, RMSE = 3.96) in repeat-doses *in vivo* data. 9^#^, 20^#^, 97^#^, and 40^#^ are acetaminophen, captopril, quinidine, and disopyramide, respectively. Purple color and yellow color in this figure indicate data from the single-dose and repeat-doses systems, respectively.

**Figure 4:**
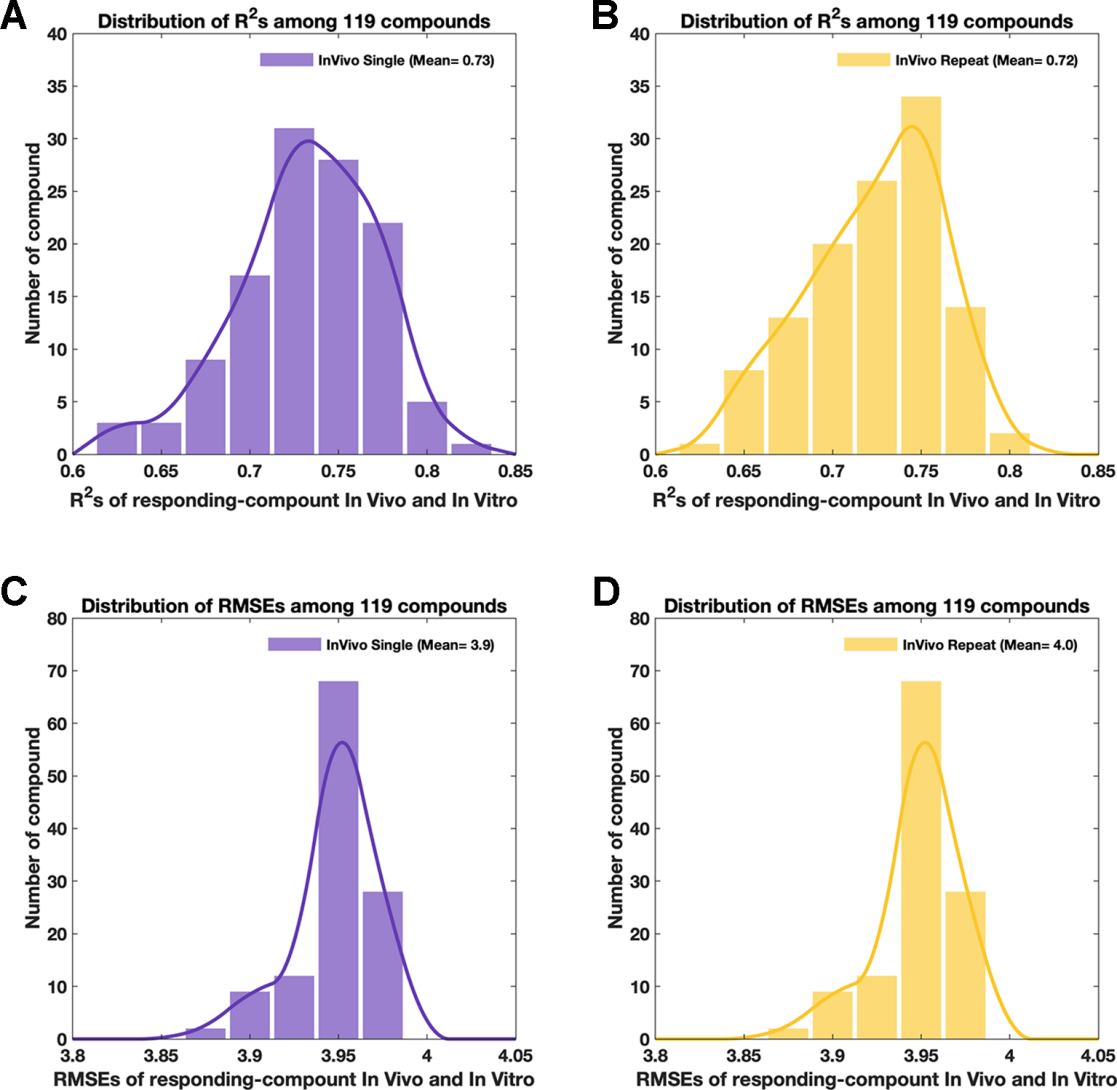
The attributions of simulated *in vivo* data. Purple color and yellow color in this figure always indicate data from the single-dose and repeat-doses systems, respectively. **(A** and **B)** The distribution of R^2^ among 119 compounds between the original *in vivo* and the simulated *in vivo* data for single-dose and repeat-doses data, respectively. **(C** and **D)** The distribution of RMSE among 119 compounds between the original *in vivo* and the simulated *in vivo* data for single-dose and repeat-doses data, respectively.

The consistency of the “drug-responding component” and *in vitro* was already confirmed in the validation stage by using the PRank score and Dice’s coefficient. However, when replacing (swapping) the “drug-responding component” with *in vitro* data, consistency at the genetic quantification level still needs to be verified to ensure this replacement was feasible. Thus, we investigated the consistency of the “drug-responding component” with *in vitro* data gene by gene for each compound (**Figures 3** and **4**). For each compound, out of 119 compounds, we used gene expression profiles from the “drug-responding component” and *in vitro* data to plot the intensity scatter, then calculated the R^2^ and root-mean-square error (RMSE) for evaluation. Each scatter showed the corresponding tendencies of an expressed gene from the “drug-responding component” and *in vitro* profiles.

Both single-dose data and repeat-doses data showed good consistency at the genetic level; most of the dots were arranged around the diagonal line in the intensity scatter chart (**Figures 3A–D**). For single-dose *in vivo* data, the average of R^2^ was 0.73, R^2^ (**Figure 4A**) ranged from 0.63–0.81, and the average RMSE (**Figure 4B**) reached 3.95. For repeat-doses *in vivo* data, the mean R^2^ reached 0.72, R^2^ ranged from 0.62–0.80, and the average RMSE was 4.00. Furthermore, the linear regression process could be applied based on the “drug-responding component” and *in vitro* data to obtain a better correlation if necessary (discussed in *How to Improve the Consistency Between In Vitro and “Drug-Responding Component” Data In Vivo*).

### *In Vivo* Data Can Be Simulated Better by Combining *In Vitro* Data With “Environmental Component”

**Figure 5:**
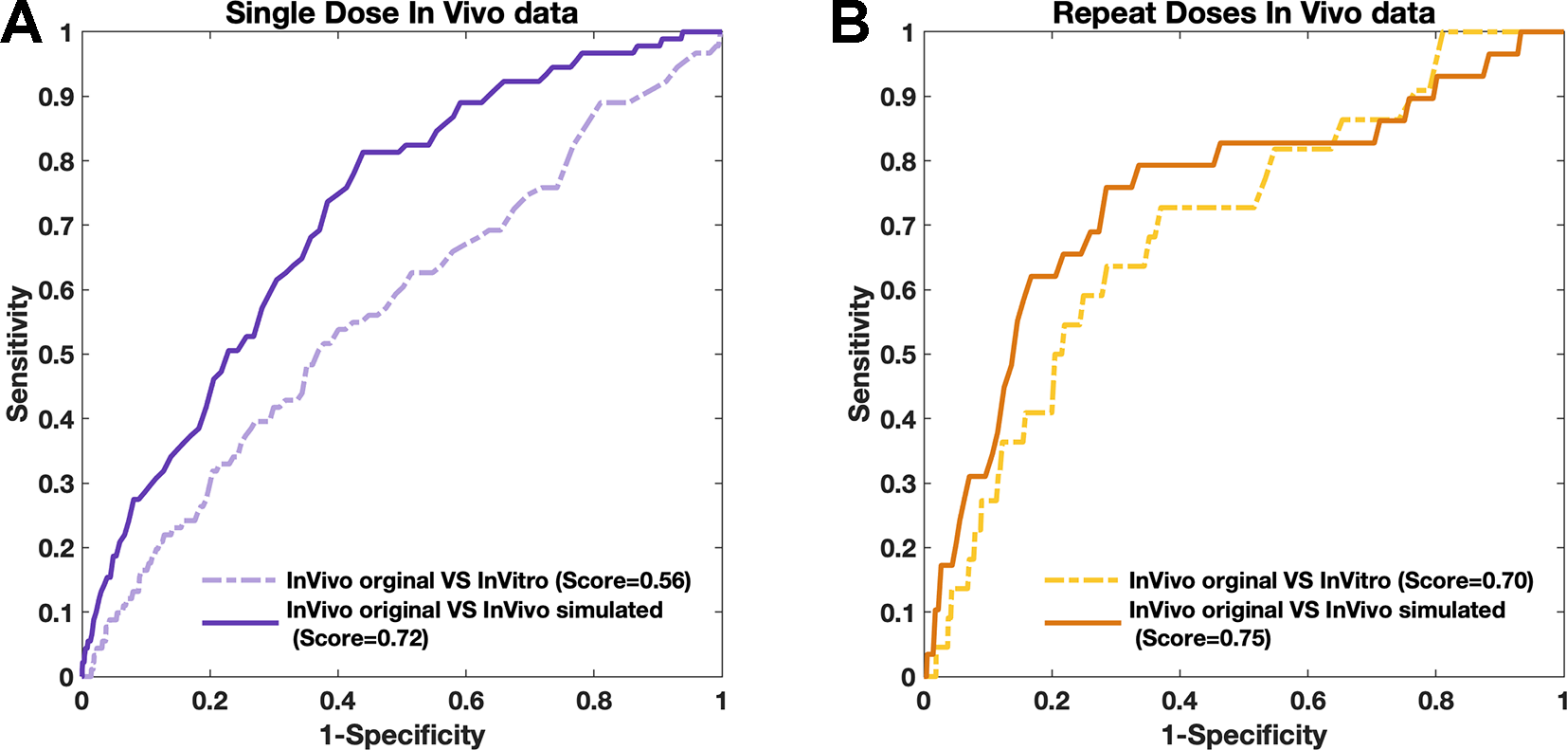
The similarities of simulated data with real *in vivo* data obtained by PRank score. **(A** and **B)** The consistencies obtained by single-dose data (purple) and repeat-doses data (yellow), respectively.

After confirming that “drug-responding component” was replaceable by using *in vitro* data, we determined that combining the correct factor with upcoming *in vitro* data allowed the simulated *in vivo* data to be obtained based on *in vitro* assays without carrying out *in vivo* assays. Since the combined data (simulated *in vivo* data) took the inner-environment into consideration, the simulation demonstrated higher compatibility with the original *in vivo* data than using the *in vitro* data alone (**Figure 5**). The “environmental component” was combined with the corresponding *in vitro* data (details are shown in the *Methods* section) for each compound. Later, the PRank score was applied again to investigate the correlation between simulated *in vivo* data and original *in vivo* data.

For both single-dose and repeat-doses *in vivo* data, the PRank scores were generated based on 7,021 compound-pairs, and high consistencies were observed, with similarity PRank scores higher than 0.72 (**Figures 5A**, **B**). The PRank score between the simulated single-dose data *in vivo* and the original single-dose *in vivo* data was 0.72 *versus* only 0.56 for the *in vitro* data and the single-dose *in vivo* data. For repeat-doses *in vivo* data, the PRank score was 0.70 when directly comparing the *in vitro* data with the original *in vivo* data and rose to 0.75 when comparing the simulated *in vivo data* with the original *in vivo* data. These results indicated that the simulated *in vivo* data had higher consistency with real *in vivo* data than did the *in vitro* data with real *in vivo* data, especially for single-dose *in vivo* systems (discussed in *Extension of Strategy Application*). Furthermore, once the attribution of two components has been confirmed, we not only can use the “drug-responding component” to obtain a better understanding of the mechanism for the drug effect but also use the “environmental component” to make the *in vitro* data become more accessible for real situations in living objects. With the storage of the “environmental component,” the upcoming *in vitro* data can always be adjusted and turned into simulated *in vivo* data.

### Validation of the Deconvolution Strategy

To further investigate the utilization of the strategy in this study, we applied the strategy to another individual dataset, which including 15 common compounds. Factor analysis also applied to validation dataset, two factors observed from *in vivo* data set and only one main factor observed from the *in vitro* data (**Figure S2**). Thus, the number of factors we used at post-modified NMF of validation is 2.

Similar to the results that observed from using TG-GATEs, when comparing original *in vivo* data with *in vitro* directly, the PRank score is 0.44 (discussed in *Further Perspective and Challenges*). And the Prank score raised to 0.50 when comparing drug-responding component from *in vivo* data with *in vitro* data. Later, simulated *in vivo* data obtained from validated dataset showed higher similarity (Prank score = 0.68) than the original comparison. Notably, after applying the linear regression, PRank score between original *in vivo* data and simulated data reached to 0.77 (discussed in *How to Improve the Consistency Between In Vitro and “Drug-Responding Component” Data In Vivo*) (**Figure S3**). The results implied that, when dealing with the data generated from different platforms (GPL341 VS GPL1355) and at different times (2008 VS 2018), the linear regression is necessary.

All in all, the performance evaluation we have shown above is positive for single-dose *in vivo* data and repeat-doses *in vivo* data in the verification stage and simulation stage, especially for single-dose systems (discussed in *Extension of Strategy Application*). The “drug-responding component” deconvoluted from *in vivo* data achieved higher similarities than the original *in vivo* data when compared with *in vitro* data. The two factors, the “drug-responding component” and the “environmental component,” generated from the original *in vivo* data were able to reveal different drug-interaction and inner-environment functions inside living bodies at the gene expression level. Additionally, this result showed more potential for extending the usage of post-modified NMF to apply to heterogeneous fields, which include the cancer clinical data that we discussed in a previous study (Liu et al., 2018a) and the large-scale toxicogenomics field that we utilized in this study.

## Discussion

### Confirm the *Number of Factors* (*k* Value) for Deconvoluting by Factor Analysis

By using factor analysis for *in vivo* data, both single-dose and repeat-doses data showed (**Figure S1**) that there were two main factors for a gene expression profile, whereas *in vitro* data only had one factor. Additionally, in terms of the factors of *in vivo* single-dose data, the first factor had a higher score than the second factor. On the other hand, the two main factors for *in vivo* repeat-doses data had almost the same score. This result suggests the second factor might be “environmental component.” Since a compound remains in the repeat-doses *in vivo* data situation for a longer time, the inner-environment would influence the data for repeat-doses more than it influences single-dose *in vivo* data.

Moreover, we confirmed that the number of main factors is 1 for *in vitro* data and 2 for *in vivo* data. As shown in **Figure S1-a**, although there are some minor factors behind the highest one or two columns, the cut-off already contributes more than 99% of factors **Figure S1-b**, specifically 99.61% for the first factor of the *in vitro* data and 99.56% and 99.65% for the first and second factors of the single-dose and repeat-doses *in vivo* data, respectively. Thus, we used k = 2 when we applied post-modified NMF for deconvolution.

### How to Improve the Consistency Between *In Vitro* and “Drug-Responding Component” Data *In Vivo*

Technically, if more samples are involved, higher accuracy can be generated during formulization. However, within the Open TG-GATEs database, for each compound at a different dose and different duration, there are three biological repetitions for *in vivo* data and two biological repetitions for *in vitro* data. The amount of sample that we can utilize in this study was large but still limited. Although this database is capable of helping us prove our verification and launch the simulation, the number of samples is far from sufficient. To pursue better performance and durability, the number of *in vivo* samples used to obtain the “drug-responding component” and the “environmental component” should be as high as possible. Additionally, once the two components have been obtained, infinite *in vitro* data can always be modified and further optimized based on those two components.

Notably, in the Open TG-GATEs database, all the data were generated by the same microarray chip (Affymetrix Support by Product for GeneChip^®^ Rat Genome 230 2.0 Array) in the same relative location. Only minor improvements for single-dose data (0.72–0.72) and repeat-doses data (0.75–0.76) (**Figure S4-a** and **b**) were achieved by applying linear regression in this study, which indicated that the data we used in this study were similar in their order of magnitude. As for the validation stage, applying linear regression has improved 9% (from 0.68 to 0.77) on PRank score than that directly combine *in vivo* data with *in vitro* data. For further simulation processing, the “environmental component” might be combined with the upcoming *in vitro* data issued from other batches, other laboratories or even other chips. The expression level of the “environmental component” might be influenced by the experimental batch. Therefore, applying linear regression is suggested to reduce potential bias at the numerical and batch effect levels.

Linear regression (y = Ax + B, A is the slope and B is the intercept) was based on “drug-responding component” (y) and *in vitro* data (x) to correct the bias at the numerical and batch effect levels between *in vitro* and *in vivo* systems. This study also demonstrated the distributions of slope and intercept for each compound to investigate the correlation between *in vivo* “drug-responding component” and *in vitro* data. Most of the slopes were in the range of 0.8–0.9 in both the single-dose and repeat-doses data (**Figure S4 c**), and the average slopes for single-dose and repeat-doses *in vivo* data were both 0.86. Additionally, the average intercepts for single-dose and repeat-doses data were 0.31 and 0.35, respectively (**Figure S4 d**). Most of the intercepts had positive values, showing that the data obtained *in vitro* had slightly higher measurements overall than corresponding the “drug-responding component” *in vivo* data. This result indicated that, compared with the “drug-responding component,” the concentration of the corresponding compound was attenuated by biological processes *in vivo*, and this bias was observed at the gene expression level by using our strategy.

### Repeat-Doses Data Achieved Better Similarity Than Single-Dose Data

According to previous reports, when comparing single-dose data (24 h) and repeat-doses data (28 days) from an *in vivo* assay, there was a high concordance between the two *in vivo* assay systems, indicating the potential to use a short-term *in vivo* assay for some endpoints, saving time and money (Liu et al., 2018b). However, for comparisons between *in vitro* and *in vivo* data, the *in vitro* TGx data set had a relatively higher similarity to the repeat-doses *in vivo* data (0.70) than did the single-doses *in vivo* data (0.56), suggesting better correlation of the *in vitro* assays with the longer-term *in vivo* assays. Specifically, gene activities associated with the survival of hepatocytes reflect a level of adaptation that resembles those under 28-day repeated dosing conditions. One explanation could be that 24 h (single dose) *in vivo* is simply the time frame in which the liver responds to a new chemical stressor and the inner-environment sways the expression level more than drug effect. Conversely, after 28 days (repeat doses) of *in vivo* treatment, there is a greater drug effect on the expression level with multiple treatments, making repeat doses more equivalent to the response of hepatocytes in cell culture (*in vitro* assay). These differences can also be observed by the factor analysis procedure (**Figure S1**); the two main factors generated by a repeat-doses data set were more even than the factors generated by a single-dose data set. Notably, after applying the simulation strategy, the similarities of the simulated data generated from the *in vitro* data set with single-dose and repeat-doses *in vivo* data were improved 16% and 5%, respectively. This result indicated that the deconvolution strategy can be an efficient way to improve IVIVE, especially in terms of assisting with the utility of short-term *in vivo* assays.

### Extension of Strategy Application

Technically speaking, all kinds of data (kidney, liver *etc.*) that meet follow criteria can be processed by the deconvoluting strategy in this study: a. There are normal samples as blank control (to identify drug effect and inner-environmental components respectively). b. The number of samples is bigger than the number of components (in this study is 2). c. The *in vitro* data which is going to participate the simulation, is generated by applying same chemical compound. Additionally, same sequencer (platform), and post-processing of *in vivo* and *in vitro* data are highly recommended.

In this study, the data involved a large number of different compounds and treatment durations, also applied to relatively small dataset (validation), but we did not find any available sequencing data that matched our study strategy. The lack of large-scale sequencing data in this context makes this struggling for has not demonstrated an approach for sequencing data. However, we performed RNA sequencing to prove the stability and reliability of post-modified NMF in our previous study, which contained sequencing data obtained from multiple human tissues and human cancers (Liu et al., 2018a). This strategy has promising potential to be utilized with toxicogenomic sequencing data in the future. We will continue collecting related data and try to apply the strategy to sequencing data once we find an appropriate and available dataset. Additionally, it is reasonable to believe that with the development of emerging genetic technologies, the strategy that we proposed might be able to utilize miRNA, ncRNA, and other kinds of genetic data.

### Further Perspective and Challenges

In this study, a computational strategy is presented to improve IVIVE problem *in silico*. The strategy is able to extract drug effect and inner-environmental components from the original *in vivo* data, further by combining *in vitro* data with inner-environmental component to narrow the gap between two experiential systems. Moreover, by keeping inner-environmental components that been obtained, more simulations can be perform with upcoming *in vitro* data.

Nevertheless, several directions still remain to investigate. As for NMF algorithm which is the focus of this study, reference-free is an advantage for NMF but also can be regarded as a delicate factor during iterations. Iterations need to avoid local optimize and also to guarantee the stability and robustness (*Post-Modified NMF Deconvolution Method*). For biological part, as we mentioned, due to conventions at processing, the number of replications of samples is lacking for mathematical calculation. And the quality control of batch affect, environmental difference, multi-platform operations are also need to count into consideration.

For this study, the different inter-lab and different inter-platform problem still needs to be further discussed. Thus, for the 119 compounds and 15 compounds that we performed in application and validation parts, respectively, we shared tables for the “environmental component” ($W_{env}^{*}$), and the average of the corresponding weight matrix ($H_{invivo}^{*}$) obtained in this study for further research in the supplementary materials (**Tables S3**–**S5**). Therefore, if any available *in vitro* data generated with the corresponding compounds is included in this study, the list can be utilized to transfer the *in vitro* data into simulated *in vivo* data (details shown in *Generation of Simulated In Vivo Data Based on In Vitro Data*). Additionally, we believe that with the utilization of our strategy, the variation caused by inter-lab and inter-platform situations can be further investigated.

## Materials and Methods

### Materials

#### Toxicogenomics Database

The rat data were downloaded from a large-scale toxicogenomics database named Open TG-GATEs (Open Toxicogenomics Project-Genomics Assisted Toxicity Evaluation Systems, http://toxico.nibiohn.go.jp/english/) (Uehara et al., 2010; Igarashi et al., 2014). This is a database that stores gene expression profiles and traditional toxicological information obtained *in vivo* (rat) and *in vitro* (primary rat hepatocytes). In total, 170 compounds with multiple doses, measure-times, and treatment durations were involved. In more detail, there are two kinds of treatment durations for *in vivo* data: one treatment duration is a single-dose trial, and the other treatment durations is a repeat-doses trail. Three systems in this study specifically included gene expression data obtained *in vitro* and *in vivo* (single dose/repeat doses). In other words, each compound involved *in vitro* data, single-dose *in vivo* data, and repeat-doses *in vivo* data.

For each compound, the *in vitro* data consisted of three doses (low, medium, and high) and three treatment time points (2, 4, and 24 h). The *in vivo* data were obtained from adult rats that were 6 weeks old. For single-dose data, the rats were treated with three doses (low, medium, and high), and liver tissue was collected at four time points (3, 6, 9, and 24 h) after treatment. For *in vivo* repeat-doses data, the rats were treated with three doses (low, medium, and high) and with different treatment durations (3, 7, 14, and 28 days). After the last exposure and dose the animals were killed 24 h later and that liver tissue was collected and isolated. Every time point (or duration) had corresponding control samples.

To reduce the influence of irrelevant variables on the measurements to guarantee external validity, the data with the highest doses, longest timepoints, and longest durations, as well as their corresponding control data, were used for our investigation. Specifically, the “*in vitro*” data are *in vitro* data that were obtained with the high dose at 24 h, “*in vivo* single” data are single-dose *in vivo* data that were obtained with the high dose at 24 h, and “*in vivo* repeat” are the repeat-doses *in vivo* data generated under the high dose at 28 days. A total of 24,023 biological samples can be utilized in Open TG-GATEs. Based on 119 common compounds (**Table S1**), 7,021 pairwise combinations for compounds were generated.

#### The Generation of Gene Expression Profiles

For application data, the microarray data downloaded from Open TG-GATEs were processed by Factor analysis for Robust Microarray Summarization (FARMS) (Hochreiter et al., 2006). For each compound, there were three and two replicate samples for *in vivo* and *in vitro* data for every time point, respectively. Every dosing sample had its matched control samples as well. Specifically, there were three control samples *in vivo* and two control samples *in vitro* for each time point for each compound. After quantile normalization of the probe-level data, we calculated the probe intensity ratios by referencing the corresponding control measurement for the blank cell culture (without compound) to correct the compound batch. Next, the probe-level names were transformed into their corresponding gene-level names by using a CDF (Version 15.1.0, ENTREZG) file (Dai et al., 2005). Later, in order to obtain expression values per gene, intensity ratios at the probe set level were summarized.

The original *in vivo* single-dose data were stored as a matrix 13,934 rows and 715 columns, and each column represented the group of 13,934 gene expression profiles for one biological sample. Similarly, the *in vivo* repeat-doses data were a matrix with 13,934 rows and 706 columns (control samples in repeat doses of some compounds had no biological repetition), and *in vitro* data were 13,934 rows and 476 columns. We marked the gene expression profiles of the *in vitro* data, single-dose *in vivo* data, and repeat-doses *in vivo* data as “*in vitro*,” “*in vivo* single,” and “*in vivo* repeat,” respectively.

#### Validation Datasets

The validation rat data contains two microarray datasets from different years and platforms, and can be both downloaded from NCBI-GEO database (Barrett et al., 2012). The *in vivo* data is obtained from data set (GSE68110) at 2008 (Ellinger-Ziegelbauer et al., 2008) on Affymetrix Rat Expression 230A Array platform. 30 compounds included with different dosages (low/middle/high). And the *in vitro* data set (GSE119933) was generated coordinate with *in vivo* at 2018 (Grinberg et al., 2018) on Rat Genome 230 2.0 array, 29 compounds with different treatment durations (1d/3d/7d/14d) are involved. Similar to the usage of GT_GATE, only the compounds that have control samples and at the highest dosage or longest treatment would be utilized for this study. That is, samples with high dosage group from *in vivo* data, and 14d treatment from *in vitro* data are involved in this study (**Table S2**).

Data preprocessing and all subsequent analyses were performed using MATLAB as well. After filtering, 15 common compounds (**Table S2**) are selected. Each compound has three experimental samples with three controls for *in vivo* and *in vitro* data, respectively. And 10324 common genes left after overlapping two platforms, 105 pairwise combinations for compounds were generated.

### Methods

#### Decision of the Number of Main Factors

Factor analysis was first released by J. Pearson (Holgado–Tello et al., 2010) and C. Spearman (Woolley et al., 2010) to determine the number of species; then, R. M. Wallace further added the matrix rank method to address multicomponent systems (Wallace, 1960; Wallace and Katz, 1964). We used factor analysis to decide upon the number of main factors in our study (discussed in *Confirm the Number of Factors (k Value) for Deconvoluting by Factor Analysis*). This statistical method (Bartholomew et al., 2008) is able to describe a group of main components generated among observed, correlated variables. Components can be regarded as several factors that potentially can lower the number of unobserved variables and maintain the main characters of the variables in the meanwhile. For example, in our case, it is possible that variations in multiple observed variables mainly reflect the variations in two underlying variables. Factor analysis searches for such joint factors in response to unobserved latent variables. Hence, factor analysis helps to deal with data sets in which there are large numbers of observed variables that can be reflected by a smaller number of latent variables.

Factor analysis was first used in psychometrics and then commonly used in chemistry (Subbarao et al., 1996), biology (Meng et al., 2011; Love et al., 2004), personality theories (Ford et al., 1986; Cattell, 1987; Kahn, 2006), and marketing (Stewart, 1981; Churchill and Iacobucci, 2006; Polit and Beck, 2008). Factor analysis can compress large data to achieve higher data quality, investigate a significant explanation, and simplify completed problems. The original data matrix is marked as *V.* By recombining observed variables linearly, the original matrix *V* can be represented with a group of new underlying variables. To generate the factors of *V,* the covariance matrices *Z* is calculated by *V.*

(1) $$Z=V^{\prime }V$$

The size of *V* is *r × c*; then, the size of *Z* is *c × c*. Then, we diagonalize the covariance matrix *Z*, and the diagonalized matrix λ is generated.

(2) $$Q^{-1}ZQ=\left[\begin{array}{c} \begin{array}{c} \lambda_{1},0,\ldots ,0\\ 0,\lambda_{2},\ldots ,0 \end{array}\\ \ldots \\ 0,0,\ldots ,\lambda_{c} \end{array}\right]=\lambda$$

Q=[q_1_,q_2_,…,q_c_] is the matrix consisting of eigenvectors and meeting the orthogonality (Q^−1^ = Q^τ^, the symbol “τ” in formulas represents the transposition of the corresponding matrix), Q^−1^ is the inverse matrix of Q, and *λ_i_* is the eigenvalue that meets the criteria:

(3) $${\text{Zq}}_{j}=\lambda_{i}q_{j}\;,\;where\;j=1,\ldots ,c.$$

Then, we can utilize λ to calculate reduced eigen value (REV) based on *V* by the following equation:

(4) $${\text{REV}}_{j}=\lambda_{j}/\left(\text{r}-\text{j}+1\right)\left(\text{c}-\text{j}+1\right)$$

Furthermore, a list of ratios could be derived by the REVs:

(5) $${\text{ratio}}_{\text{t}}=\frac{REV_{t}}{REV_{t+1}},\;where\;t=1\ldots j-1.$$

Eventually, the maximum ratio can be regarded as the most significant factor, and the minimum ratio can be regarded as the least significant factor. In this study, the number of factors that added more than 99% cumulative contribution were considered main factors (details shown in *Confirm the Number of Factors (k Value) for Deconvoluting by Factor Analysis*). Those main factors were able to reflect *V* and could be visualized to obtain better observations.

#### Post-Modified NMF Deconvolution Method

Non‐negative matrix factorization (NMF) is a series of unsupervised learning methods that is able to factorize a nonnegative matrix *V* into 2 nonnegative matrices (*W* and *H*) (Lee and Seung, 1999). The post-modified NMF that we used in this study is an unsupervised learning algorithm that is capable of estimating the gene expression profiles and contents of the major components in samples without any prior reference knowledge. NMF is frequently used in blind source separation because of its nonnegative conception. The ultimate objective of NMF can be described as: given a matrix *V,* NMF finds nonnegative matrices *W* and *H. W*·*H* is a lower‐rank approximation of *V.* NMF estimates the basis matrix *W* (*m × k* nonnegative matrices) and the coefficient matrix *H* (*k × n* nonnegative matrices) from the original matrix *V* (*m × n* nonnegative matrix). In practical application, the factorization rank k should meet the conditions that *k* ≪ Min (*m, n*). After deconvoluting and iterating from the original matrix *V* to the sum of the loss function F (*V, WH*) (Gaujoux and Seoighe, 2010), a regularization function F (*W, H*) is reached at the minimum, and the optimized *W* and *H* are generated (Hoyer, 2004; Kim and Park, 2007). The original NMF algorithm can be written as:

(6) V≈W⋅H

(7) $$V_{j}=\sum_{i=1}^{k}W_{j}H_{ij}\left(\;j=1,\;2,\;\ldots ,the\;number\;of\;columns\;in\;V\right)$$

The matrices W and H were estimated using an alternating least squares (ALS) algorithm, which was first proposed by Paatero and Tapper (Paatero and Tapper, 1994) and improved by Paatero and Albright et al. (Paatero, 1999). In the ALS algorithm, *W* is initialized as a random dense matrix and used to solve *H* using a least squares calculation step. The negative elements in *H* are set to 0. The loss function F (*V, WH*) is applied to measure the result of each iteration. In this function, the factors *W* and *H* are chosen to minimize the root‐mean‐squared residual *D* (the cost function) between *V* and W·H. That is, when loss function F (*W, H*) is reached at the minimum, the optimized *W* and *H* are generated. The iteration procedure is iterated until *W* and *H* can minimize the cost function *D* (Berry et al., 2007). The loss function F algorithm can be written as:

(8) $$\text{F}\left(\text{W},\text{H}\right)=\|V-WH{\|}_{F}^{2}$$

Moreover, for better biological utilization of a practical situation, a normalized H* can be generated by normalizing the *H* after deconvoluting. Specifically, by restricting the sum of the values in each column of *H* to 1, a normalized H* is generated (*k* components × *n* samples).

(9) $$\text{H}=\left[h_{1},\;h_{2},\;\ldots ,h_{j}\;\left(j\;is\;the\;number\;of\;columns\;in\;V\right)\right]$$

(10) $$H_{ij}^{*}=\frac{H_{ij}}{\sum_{l=1}^{k}H_{lj}}$$

After *H* being normalized as H*, with known H* and original matrix V, W* can be generated accordingly. Notably, as the W* might contain a tiny amount of negative values (less than 0.01% in all compounds), we set the negative values to zero to keep the matrix nonnegative. Similar to NMF, the essence of post-modified NMF (Liu et al., 2018a) is a multivariate linear model. Each column of *V* is approximately expressed as a linear combination of the column vectors in W* and the coefficient matrix H* of corresponding columns. The procedure results in a new W* as follows:

(11) $$W^{*}=\text{V}H^{*\tau }{\left(H^{*}H^{*\tau }\right)}^{-1}$$

The symbol “τ” in formulas represents the transposition of the corresponding matrix.

In our study, for each compound in every system, the matrix *V* was the original gene expression data, including the expression of *m* genes in *n* mixed samples (*m × n* matrix), where *m* represents the number of genes, and *n* represents the number of samples. W* was a deconvoluted signal matrix, including the expression profiles of *m* genes in *k* individual component‐types (*m × k* matrix), and H* was a weight matrix that included the relative weight of *k* component types in *n* mixtures (*k × n* matrix). Matrices *V*, W*, and H* were all nonnegative.

Since the algorithm for generating W* and H* needs iterations for parameter optimization, which might lead to local minima, repeated factorizations with random initial W* and H* may yields different *W* and *H* pairs, which might include less optimal results. To ensure reliable and conforming results, the deconvolution procedure was repeated 100,000 times in total for every compound. After 1,000 inner-iterations with a different initial W* and H*, to select the results with the minimum root‐mean‐squared error, yielded from (Boess et al., 2003), and then repeat the whole processing 100 times to get the average result. The averaged results were used for further analysis to ensure reliability and optimization.

After factor analysis in *Decision of the Number of Main Factors*, 2 factors were yielded from the *in vivo* data. We sought to determine which factor represents the inner-environment of the inner body and which factor represents the response of the drug effect. After deconvoluting by post-modified NMF, the matrix of the gene expression profiles of each compound for *in vivo* data was factorized into two matrices: W* represented the gene expression profiles, and H* represented the weights of two factors. We compared each column of W* with the corresponding control sample for each compound by using pairwise Pearson correlation coefficients. The column with the higher coefficient was considered the “environmental component,” while the other column with the lower coefficient from the control sample was considered the “drug-responding component.” Thus, each column of W* in formula (Cattell, 1987) (*Post-Modified NMF Deconvolution Method*) represented the estimated expression levels of *m* genes in a component, which could be compared with the corresponding expression levels of the matched control sample.

#### Identification of Biologically Significant Genes

The biologically significant genes identified between the dosing samples and control samples may be potentially correlated with phenotypic differences. For each compound in each assay system, fold change (FC) values were generated by comparing the dosing group VS the matched control group. Then, we ranked the genes from the highest FC value to the lowest, and significant genes were selected from the top and bottom of the ranked list. To find the best cut-off number of significant genes for our study, we investigated the stability of ranked FC lists of each compound by cutting at different amounts (from 50 to 550 by step 50). A stable trend appeared after 200 genes from the ranked FC list. Hence, we used 200 as the cut-off for selecting significant genes that were both up- and downregulated. Furthermore, based on the significant genes for each compound (**Figure S5**), the pairwise similarity could be calculated for PRank processing.

#### Pair Ranking (Prank) Method

The Pair Ranking (PRank) method was used to investigate the consistency among the three rat TGx assay systems (Liu et al., 2017; Liu et al., 2018b). The PRank score was utilized to measure the similar extent between every system in the validation part as well as in the application. Similar to the definition of the ROC curve, the similarity score ranged from 0 to 1, and the higher score was better.

Biologically significant genes were ranked according to their fold change value. The procedure utilized to find the threshold was the same as that in Liu’s work, and the threshold of 200 was kept afterwards. That is, biologically significant genes were obtained for each compound by finding the top and bottom 200 ranked genes by their fold change values (**Figure S3**).

Dice’s coefficient was employed to calculate the similarity between the gene expression profiles of compounds, as suggested by the SEQC I study (Wang et al., 2014; Xu et al., 2016). The pairwise compound similarity of any two compounds within a system was calculated by using the total number of 400 genes obtained for each compound. Notably, the regulated direction of overlapping genes was taken into consideration in this situation.

(12) $$\text{Dice}\prime \text{s coefficient}=\frac{N_{i,j,up}+N_{i,j,down}}{400}$$

N*_i_*,*_j_*,*_up_* and N*_i_*,*_j_*,*_down_* indicate the number of overlapping up- and downregulated genes, respectively, between compound *i* and *j*.

The pairwise similarities are ranked from highest to lowest in each system separately. Eventually, by using a receiver operating (ROC) curve and the area under the curve (AUC), the PRank score between any two systems can be calculated. To obtain the PRank score, the ranked Dice’s coefficient is transferred into a binary model (0/1) with 0.4 as the cut-off. This cut-off is close to the 95% quantile. The built-in function *perfcurve* in MATLAB R2018b was applied for ROC-AUC calculation.

#### Generation of Simulated *In Vivo* Data Based on *In Vitro* Data

After deconvolution and component confirmation of *in vivo* data, we determined that the *in vitro* gene expression profile had high similarity with the “drug-responding component” deconvoluted from the *in vivo* data. Additionally, the “environmental component” tends to reflect more attribution of the inner body. Thus, once the “environmental component” was obtained, we obtained the simulated *in vivo* data based on the *in vitro* expression profile by replacing the “drug-responding component” with the *in vitro* data. The replacement procedure can be formulized as follows:

(13) $$V_{in\;vivo}=W_{in\;vivo}^{*}H_{in\;vivo}^{*}$$

**Figure 6:**
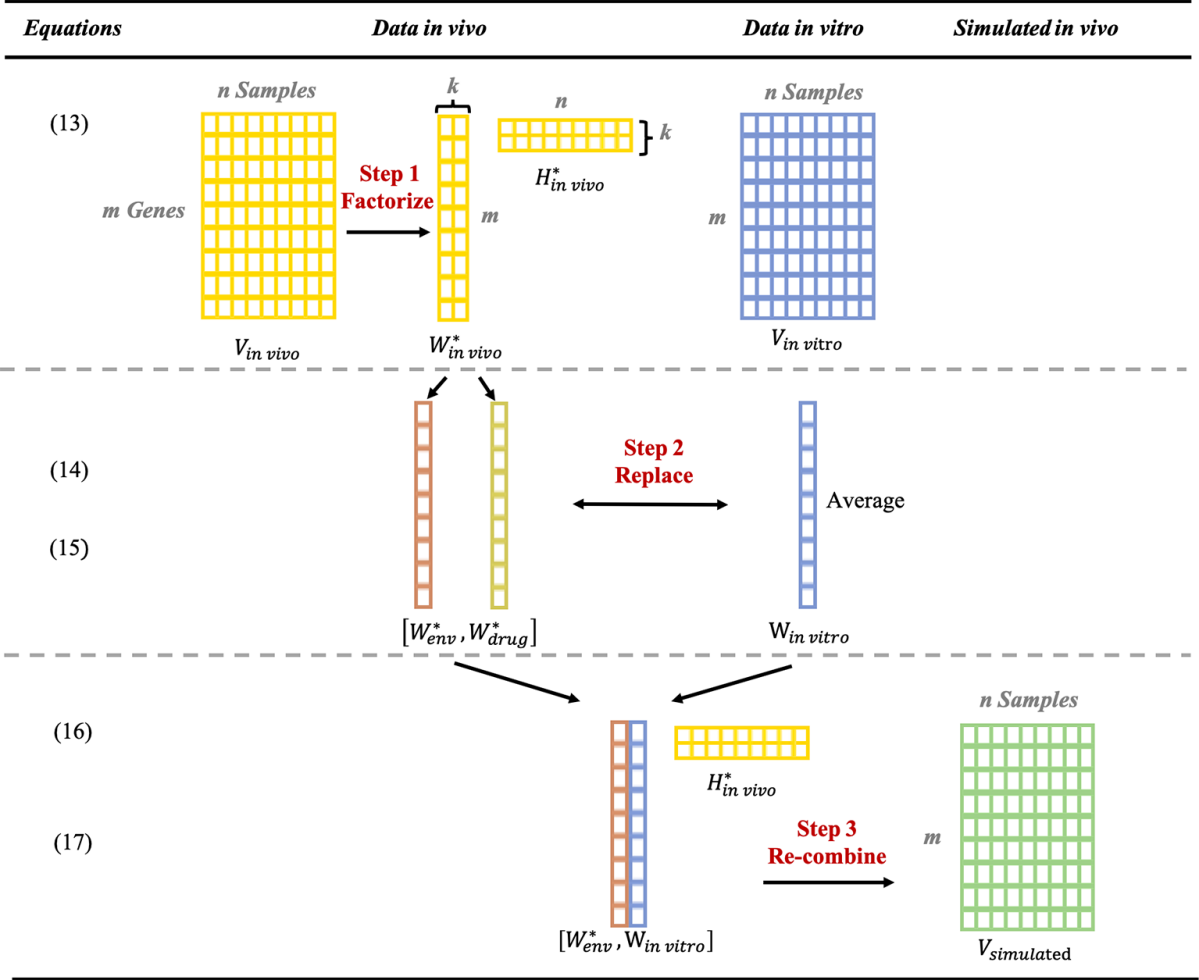
Step by step illustration of generating the simulated *in vivo* data by combining *in vitro* data with environmental component from *in vivo* data.

*V_in vivo_* is the original gene expressed profile for *in vivo* data which contains *m* genes (rows) × *n* sample (columns). The gene expression profile *V_in vivo_* is deconvoluted into $W_{in\;vivo}^{*}$ (*m × k* factors) and $H_{in\;vivo}^{*}$ (*k* factors × *n*) by using post-modified NMF (**Figure 6**). $W_{in\;vivo}^{*}$ is composed of two columns of gene expression profiles,$W_{env\;}^{*}$ and $W_{drug\;}^{*}$, corresponding to the “environmental component” and the “drug-responding component,” respectively; and $H_{in\;vivo}^{*}$ is composed of two rows of gene expression profiles, $H_{env\;}^{*}$ and $H_{drug\;}^{*}$, corresponding to the weight of “environmental component” and the “drug-responding component,” respectively; the order of these two columns depends on their similarity with matched control samples.

(14) $$W_{in\;vivo}^{*}=\left[W_{env\;}^{*},\;W_{drug}^{*}\right]$$

To prepare for replacement, the *in vitro* data *V_invivo_* is formed into the same size (1 column × the number of genes) as $W_{drug}^{*}$. The average of the *in vitro* data for each compound is utilized, which we named w *_in vivo_*.

(15) $${\text{W}}_{in\;vitro}=avg\;\left(V_{in\;vitro}\right)$$

Later, by replacing $W_{drug}^{*}$ with W*_in__vitro_*, W*_modified_*is generated.

(16) $${\text{W}}_{modified\;}=\left[W_{env\;}^{*},{\text{W}}_{in\;vitro}\right]$$

Eventually, with the recombination of w*_modified_* and $H_{in\;vivo}^{*}$, the simulated data *V_simulated_* are obtained.

(17) $$V_{simulated}=\;{\text{W}}_{modified\;}H_{in\;vivo}^{*}$$

According to (Cohen, 1992) and (Dai et al., 2006), by keeping $H_{in\;vivo}^{*}$ and $w_{env}^{*}$, the simulated data can always be obtained based on *in vitro* data that are currently available or *in vitro* data that are upcoming.

Note that if linear regression is applied to correct w*_in__vitro_* in equation (Cohen, 1992), then W*_in__vitro_* would be modified by $W_{drug}^{*}$. Thus, in this situation, $H_{in\;vivo}^{*}$ and $W_{in\;vivo}^{*}$and ($W_{env}^{*}$ and $W_{drug}^{*}$) are needed.

All calculation procedures and the identification of significant genes were conducted in MATLAB (MathWorks^®^, R2018b). In order to make Post-modified NMF and corresponding strategy easier to use, we have released a functional MATLAB package with detailed tutorial at github. (https://github.com/annlyuan/Post_modified_NMF).

## Conclusions

In this study, an *in silico* strategy based on post‐modified NMF was proposed to factorize the inner-environmental factor from *in vivo* assays at the gene expression level. Drug effect and inner-environmental components were obtained from the *in vivo* data. This strategy first verified its applicability to TGx data and then simulated the *in vivo* data by correcting the *in vitro* data. Similarities between real *in vivo* data and simulated data were higher than those obtained by directly comparing real *in vivo* data with *in vitro* data. The results indicated that this strategy could promptly generate substitutions for *in vivo* TGx. Additionally, a simulation can be generated by using *in vitro* data to reduce the launch of live-animal tests. Eventually, the gap between *in vivo* and *in vitro* data at the gene expression level is effectively narrowed.

## Data Availability Statement

Publicly available datasets were analyzed in this study. This data can be found here: https://toxico.nibiohn.go.jp/open-tggates/english/search.html.

## Author Contributions

YL conceived, designed, and performed the experiments. RJ offered ideas on quantity indicators for comparison. YL, ZW, and ML wrote the paper. YL and ZW designed the post-modified NMF algorithm that was used in the analysis. All authors discussed the results and commented on the manuscript.

## Funding

This work was supported by grants from the National Natural Science Foundation of China (No. 21675114 and No. 21575094).

## Conflict of Interest

The authors declare that the research was conducted in the absence of any commercial or financial relationships that could be construed as a potential conflict of interest.

## Supplementary Material

Figure S1 The bar graphs for the factors obtained from each system after factor analysis. **(A)** Represents the reduced eigen values (REVs) calculated by factor analysis of each factor in *in vitro* (left), single-dose *in vivo* (middle) and repeat-doses *in vivo* (right) data. **(B)** Represents the ratios of contribution calculated based on REVs in *in vitro* (left), single-dose *in vivo* (middle) and repeat-doses *in vivo* (right) data.

Figure S2 The bar graphs for the factors obtained from each system of validation data set after factor analysis. **(A)** Represents the reduced eigen values (REVs) calculated by factor analysis of each factor in *in vivo* (upper), and the ratios of contribution calculated based on REVs of *in vivo* (down) data. **(B)** Represents the reduced eigen values (REVs) calculated by factor analysis of each factor in *in vitro* (upper), and the ratios of contribution calculated based on REVs of *in vitro* (down) data.

Figure S3 Results of validation dataset. **(A)** In the comparison with original *in vivo* data (light blue), the drug-responding component achieved higher consistencies. **(B)** The comparison of applying linear regression or not. **(C)** The violin plots of the absolute difference values between *in vivo* and *in vitro* (\Dice’s coefficient of *in vivo* data - Dice’s coefficient of *in vitro* data\) of validation datasets.

Figure S4 The attributions of applying linear regression to *in vitro* and “drug-responding compound” *in vivo* data. Purple color and yellow color in this figure always indicate the data from single-dose and repeat-doses systems, respectively. **(A)** and **(B)** The consistencies between original *in vivo* data and simulated *in vivo* data obtained by PRank score. The gray lines indicate the scores obtained based on the simulated data (which are derived from applied linear regression between “drug-responding compound” and *in vitro* data) compared with original *in vivo* data. **(C)** and **(D)** The slope and intercept distributions, respectively, after launching linear regression between original *in vivo* and simulated *in vivo* data.

Figure S5 The stability of the ranked similarity list for each assay and system. The data points were calculated by comparing the ranked similarity list with different numbers of differentially expressed genes by using Spearman’s correlation coefficient.

Table S1 The list of 119 common compounds.

Table S2 The sample information of validation dataset (NA: no information available, h: hour, d: day).

Table S3 The $W_{env}^{*}$ and the average of $H_{env}^{*}$ obtained by single-dose in vivo data (format as .xlsx).

Table S4 The $W_{env}^{*}$ and the average of $H_{env}^{*}$ obtained by repeat-doses in vivo data (format as .xlsx).

Table S5 The $W_{env}^{*}$ and the average of $H_{env}^{*}$ obtained by validation in vivo data (format as .xlsx).
