# Supplementary figures and images for: Narrowing the Gap Between In Vitro and In Vivo Genetic Profiles by Deconvoluting Toxicogenomic Data In Silico
Source: Front Pharmacol. 2020 Jan 8;10:1489. doi: 10.3389/fphar.2019.01489 (PMC6964707; doi:10.3389/fphar.2019.01489)

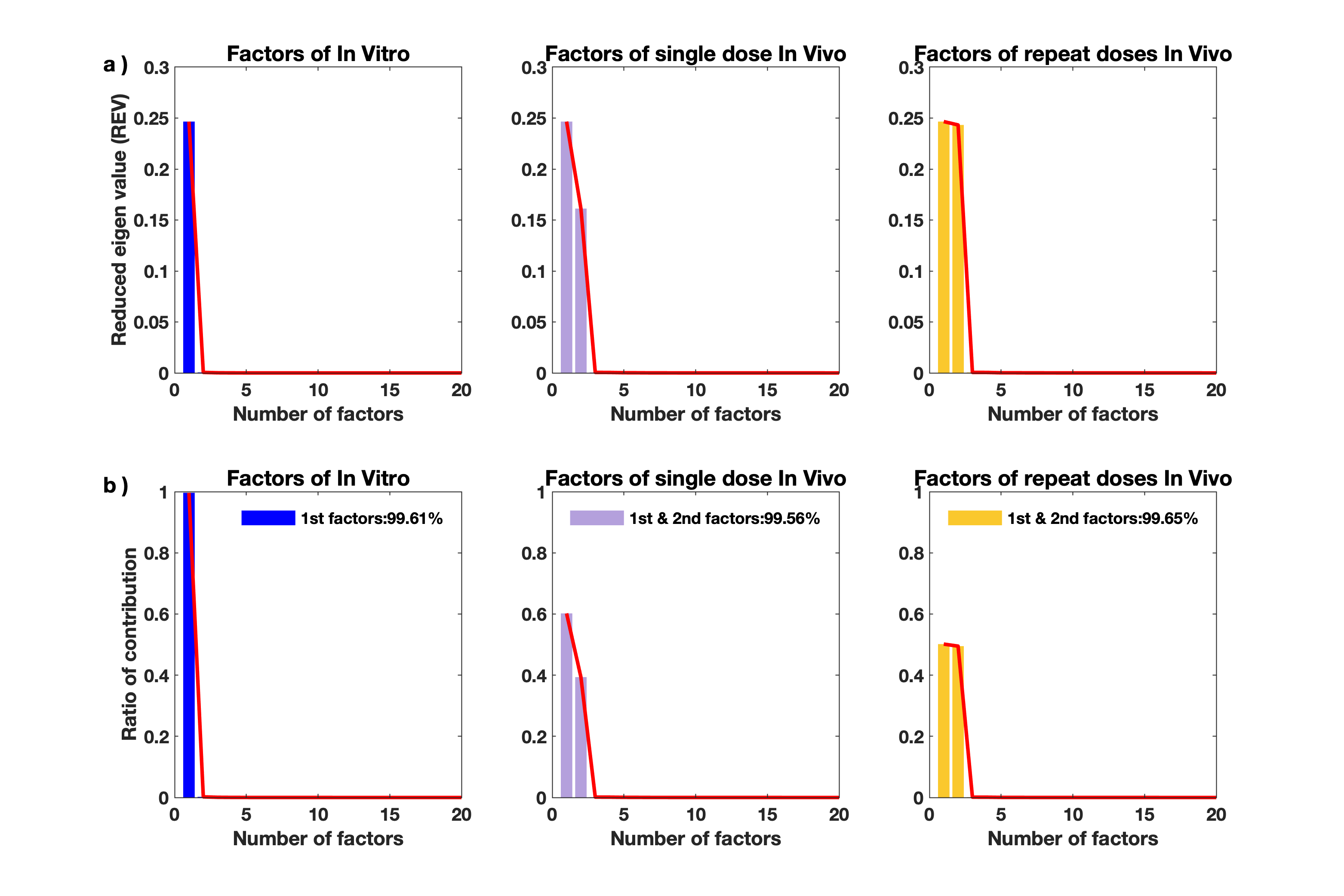

Supplement: Figure S1 — The bar graphs for the factors obtained from each system after factor analysis. (A) Represents the reduced eigen values (REVs) calculated by factor analysis of each factor in in vitro (left), single-dose in vivo (middle) and repeat-doses in vivo (right) data. (B) Represents the ratios of contribution calculated based on REVs in in vitro (left), single-dose in vivo (middle) and repeat-doses in vivo (right) data. [file Image_1.tif]

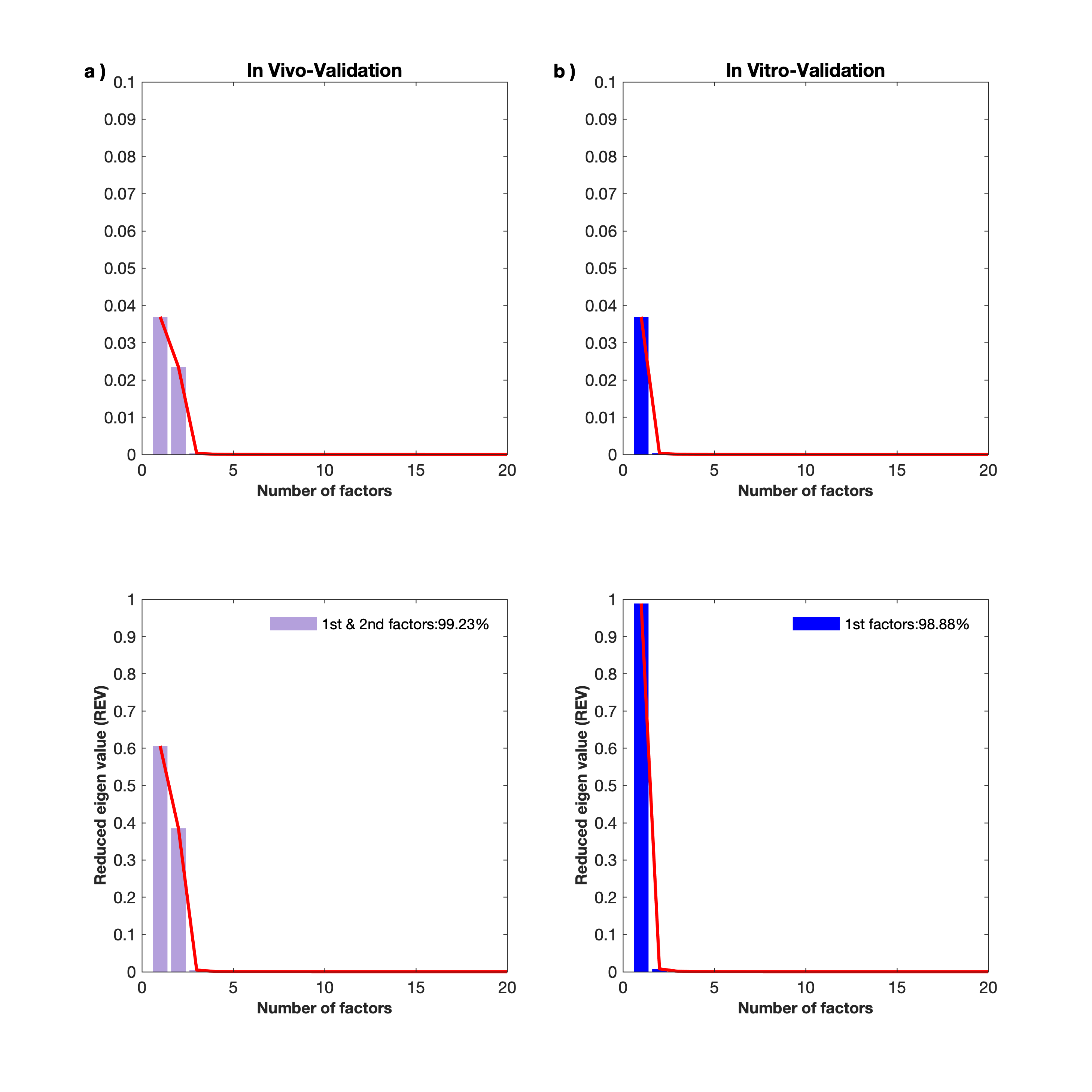

Supplement: Figure S2 — The bar graphs for the factors obtained from each system of validation data set after factor analysis. (A) Represents the reduced eigen values (REVs) calculated by factor analysis of each factor in in vivo (upper), and the ratios of contribution calculated based on REVs of in vivo (down) data. (B) Represents the reduced eigen values (REVs) calculated by factor analysis of each factor in in vitro (upper), and the ratios of contribution calculated based on REVs of in vitro (down) data. [file Image_2.tif]

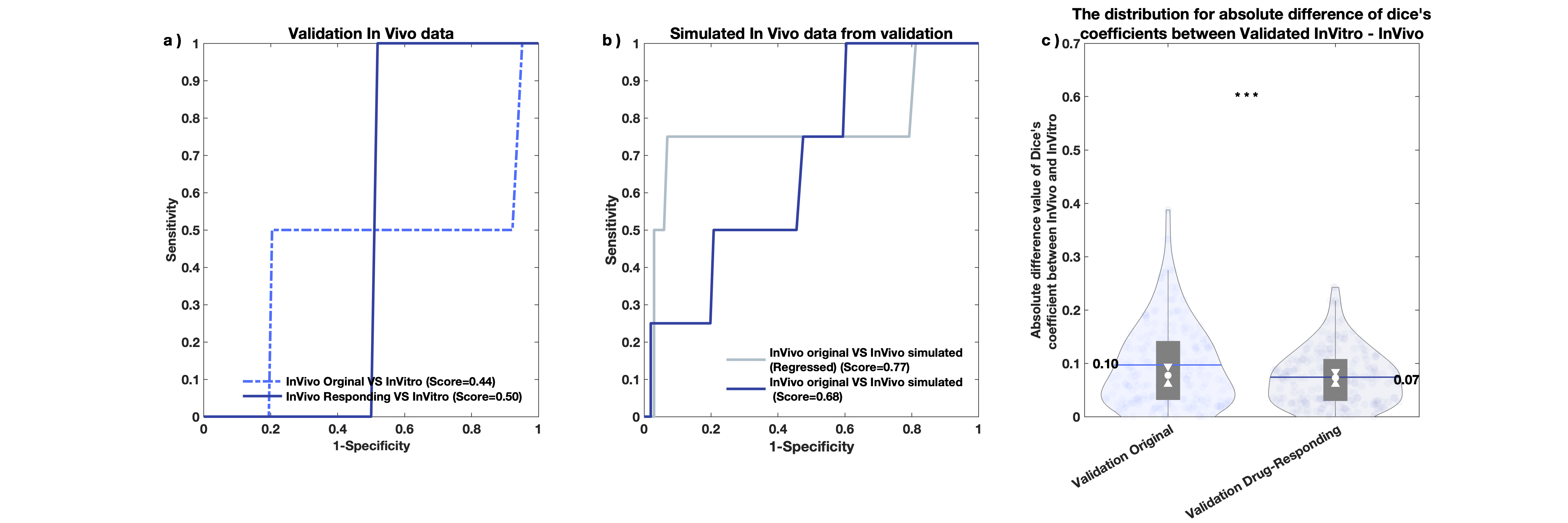

Supplement: Figure S3 — Results of validation dataset. (A) In the comparison with original in vivo data (light blue), the drug-responding component achieved higher consistencies. (B) The comparison of applying linear regression or not. (C) The violin plots of the absolute difference values between in vivo and in vitro (\Dice’s coefficient of in vivo data - Dice’s coefficient of in vitro data\) of validation datasets. [file Image_3.tif]

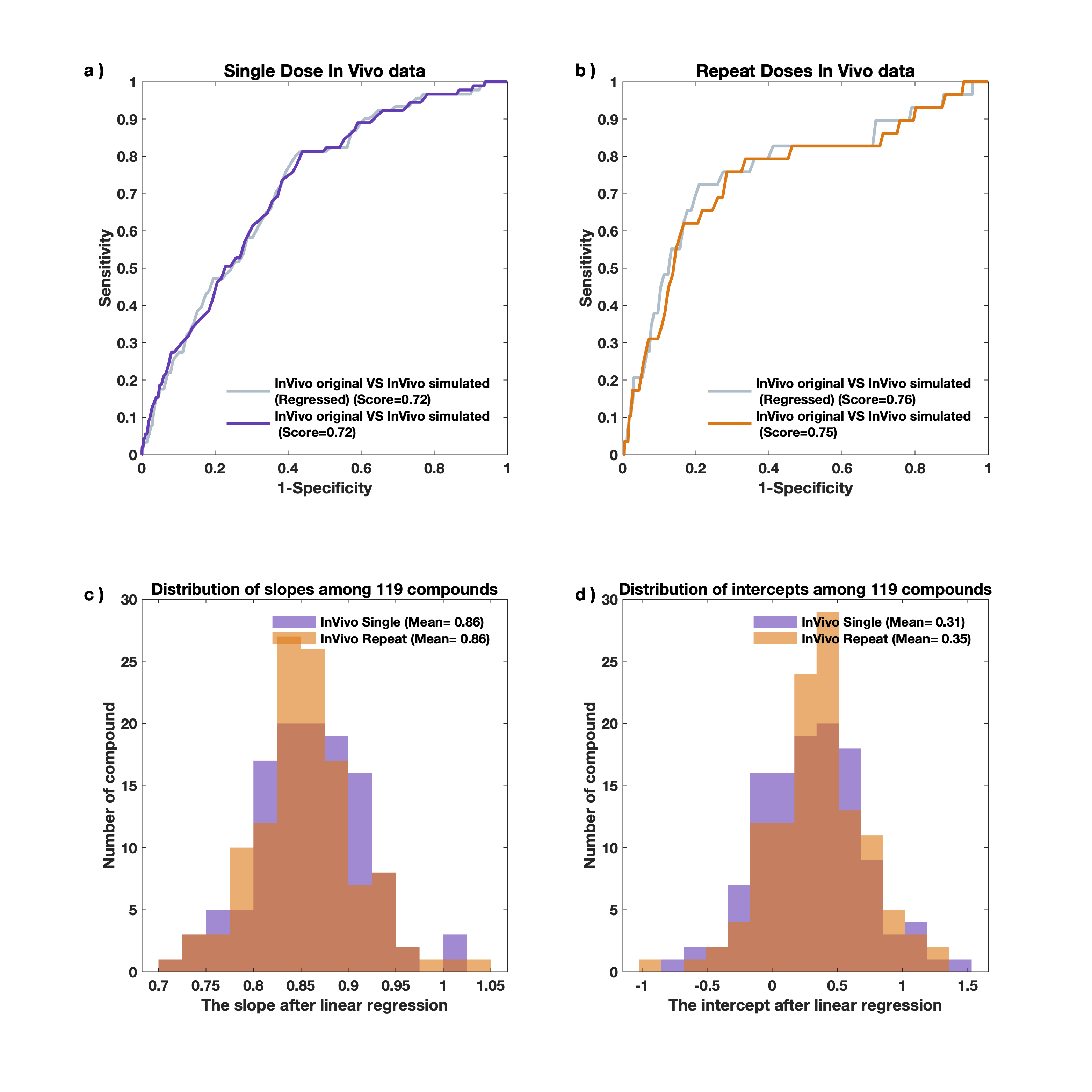

Supplement: Figure S4 — The attributions of applying linear regression to in vitro and “drug-responding compound” in vivo data. Purple color and yellow color in this figure always indicate the data from single-dose and repeat-doses systems, respectively. (A) and (B) The consistencies between original in vivo data and simulated in vivo data obtained by PRank score. The gray lines indicate the scores obtained based on the simulated data (which are derived from applied linear regression between “drug-responding compound” and in vitro data) compared with original in vivo data. (C) and (D) The slope and intercept distributions, respectively, after launching linear regression between original in vivo and simulated in vivo data. [file Image_4.tif]

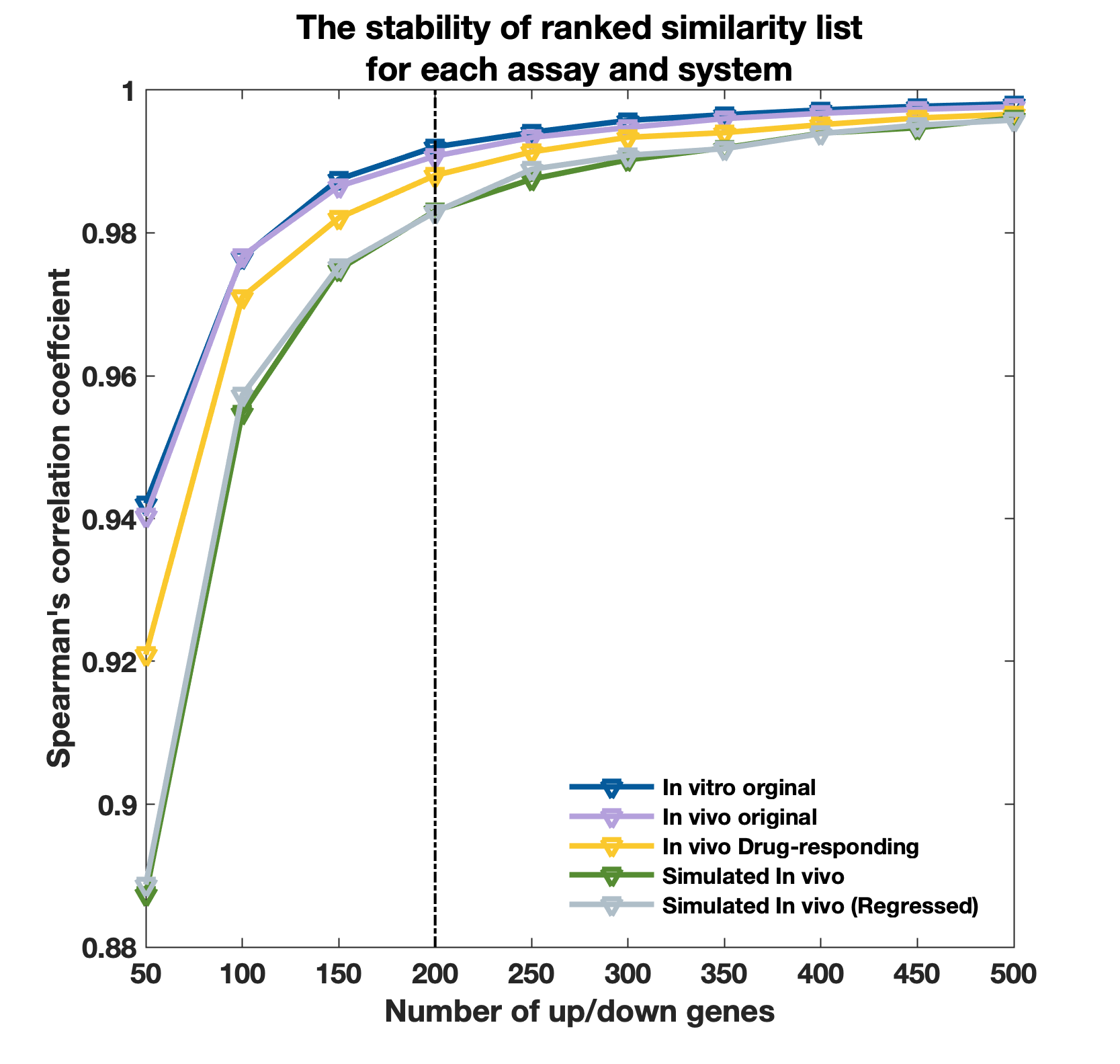

Supplement: Figure S5 — The stability of the ranked similarity list for each assay and system. The data points were calculated by comparing the ranked similarity list with different numbers of differentially expressed genes by using Spearman’s correlation coefficient. [file Image_5.tif]
