# Supplementary material for: Narrowing the Gap Between In Vitro and In Vivo Genetic Profiles by Deconvoluting Toxicogenomic Data In Silico
Source: Front Pharmacol. 2020 Jan 8;10:1489. doi: 10.3389/fphar.2019.01489 (PMC6964707; doi:10.3389/fphar.2019.01489)
Supplement: Table S1 — The list of 119 common compounds. [file DataSheet_1.docx]

# Narrowing the gap between *in vitro* and *in vivo* genetic profiles by deconvoluting toxicogenomic data *in silico*

**Yuan Liu^1^, Runyu Jing^2^, Zhining Wen^1*^, Menglong Li^1*^**

^1^College of Chemistry, Sichuan University, Chengdu, Sichuan 610064, China

^2^College of Cybersecurity, Sichuan University, Chengdu, Sichuan 610207, China

^*^ Correspondence:

Menglong Li, College of Chemistry, Sichuan University, Chengdu 610064, P.R. China.

Tel: +86-28-85412291

Email: [liml@scu.edu.cn](mailto:liml@scu.edu.cn)

Zhining Wen, College of Chemistry, Sichuan University, Chengdu 610064, P.R. China.

Tel: +86-28-85412138

Email: [w_zhining@163.com](mailto:w_zhining@163.com)

**Supplementary materials**

| **Figure S1** | The bar graphs for the factors obtained from each system of application data after factor analysis. |
| --- | --- |
| **Figure S2** | The bar graphs for the factors obtained from each system of validation data set after factor analysis. |
| **Figure S3** | Results of validation dataset |
| **Figure S4** | The attributions of applying linear regression to *in vitro* and “drug-responding compound” *in vivo* data. |
| **Figure S5** | The stability of the ranked similarity list for each assay and system. |
| **Table S1** | The list of 119 common compounds |
| **Table S2** | The sample information of validation dataset |
| **Table S3** | The $W_{\mathrm{env}}^{*}$ and the average of $H_{\mathrm{env}}^{*}$ obtained by single-dose *in vivo* data (format as *.xlsx*) |
| **Table S4** | The $W_{\mathrm{env}}^{*}$ and the average of $H_{\mathrm{env}}^{*}$ obtained by repeat-doses *in vivo* data (format as .*xlsx*) |
| **Table S5** | The $W_{env}^{*}$ and the average of $H_{env}^{*}$ obtained by validation in vivo data (format as *.xlsx*) |

**
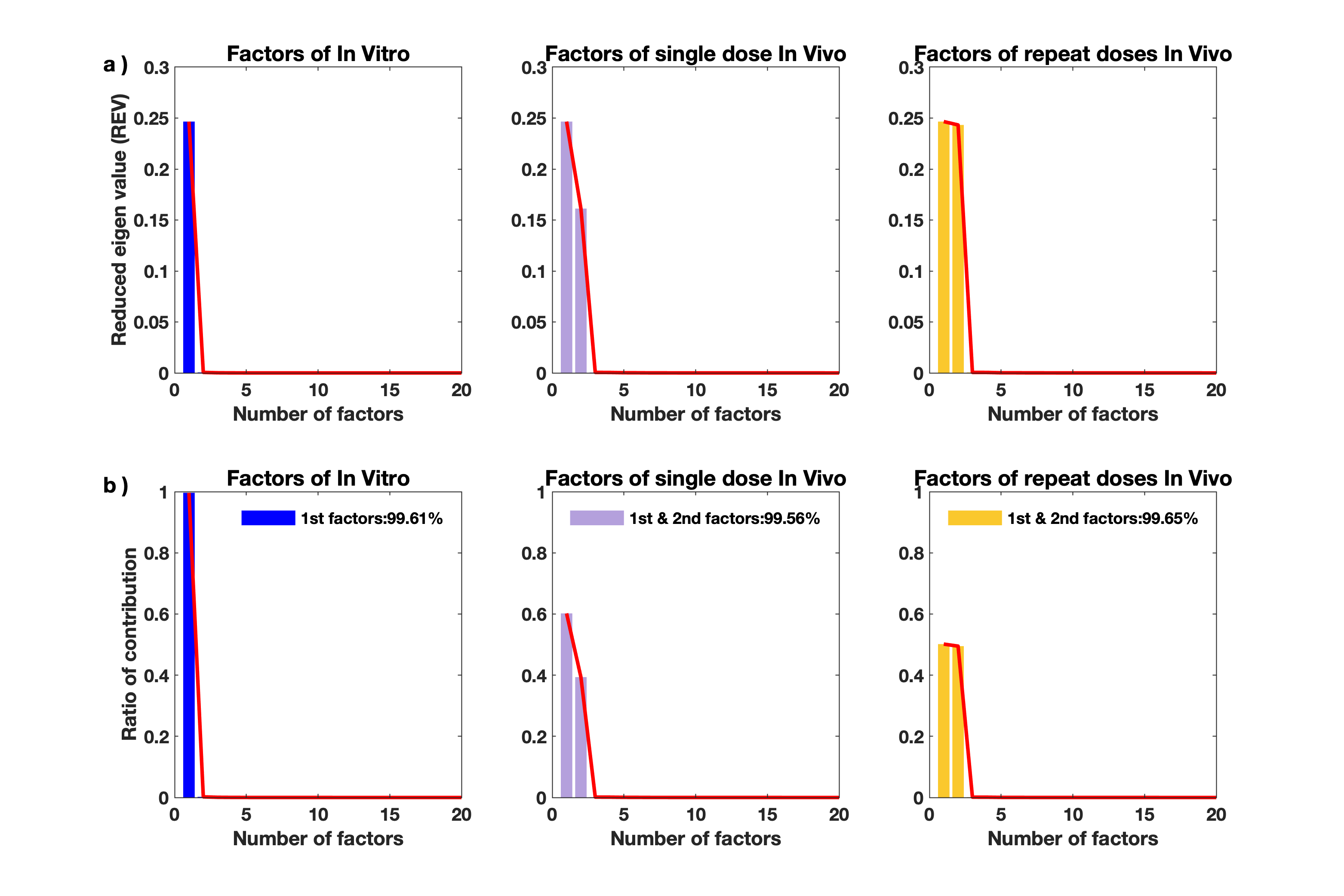
**

*Figure S1.* *The bar graphs for the factors obtained from each system after factor analysis.* *a) Represents the reduced eigen values (REVs) calculated by factor analysis of each factor in in vitro (left), single-dose in vivo (middle) and repeat-doses in vivo (right) data. b) Represents the ratios of contribution calculated based on REVs in in vitro (left), single-dose in vivo (middle) and repeat-doses in vivo (right) data.*

*
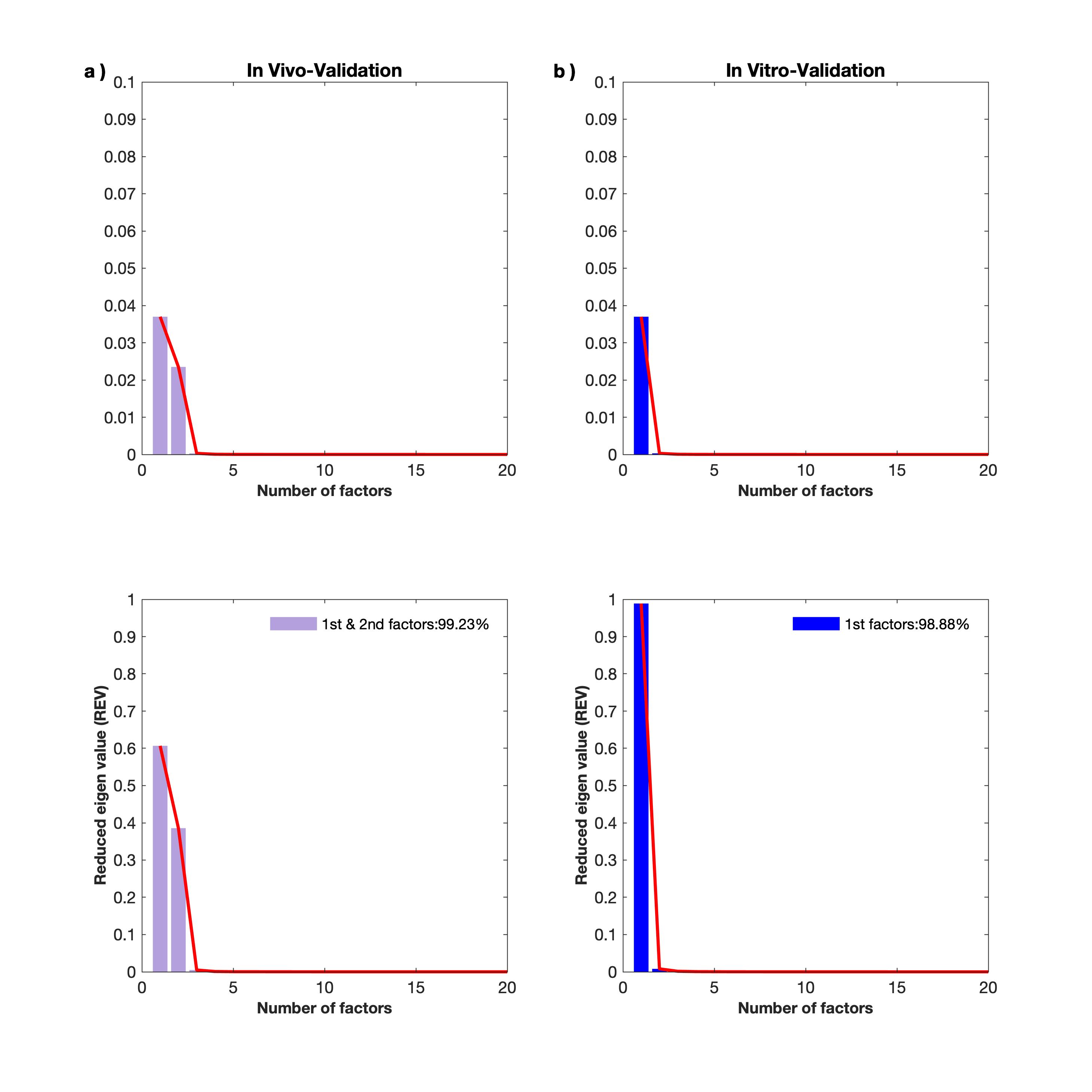
*

*Figure S2. The bar graphs for the factors obtained from each system of validation data set after factor analysis. a)* *Represents the reduced eigen values (REVs) calculated by factor analysis of each factor in in vivo（upper）, and the ratios of contribution calculated based on REVs of in vivo (down) data. b) Represents the reduced eigen values (REVs) calculated by factor analysis of each factor in in vitro（upper）, and the ratios of contribution calculated based on REVs of in vitro (down) data.*

**
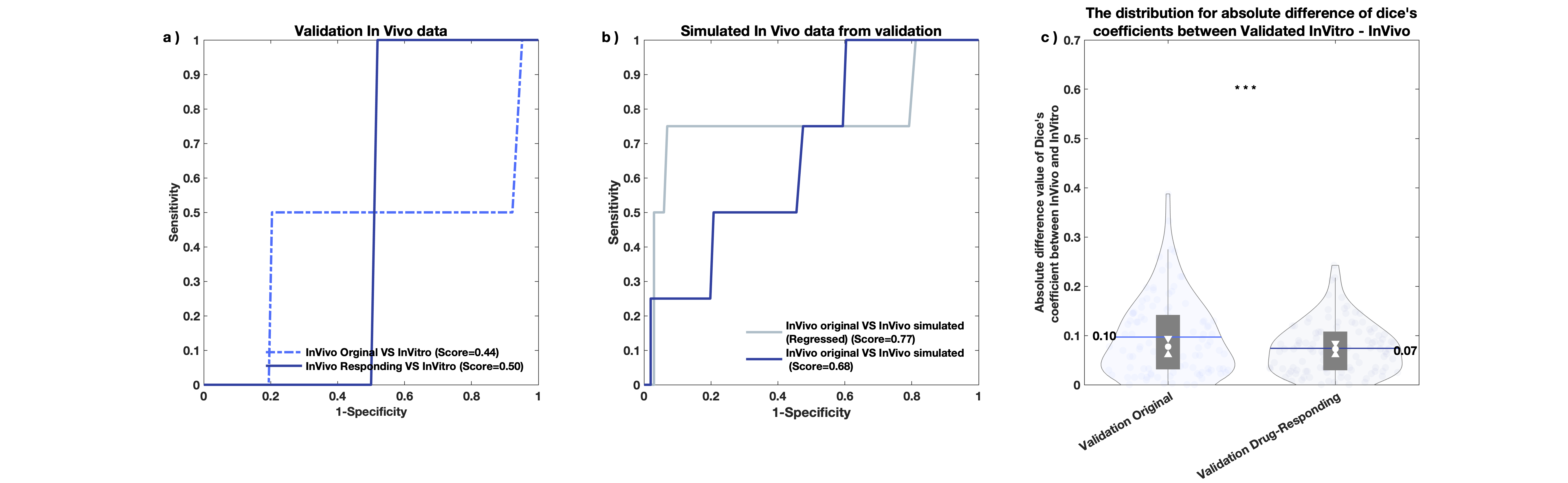
**

*Figure S3. Results of validation dataset. a) In the comparison with original in vivo data (light blue), the drug-responding component achieved higher consistencies. b) The comparison of applying linear regression or not. c) The violin plots of the absolute difference values between in vivo and in vitro (|Dice’s coefficient of in vivo data - Dice’s coefficient of in vitro data|) of validation datasets.*

**
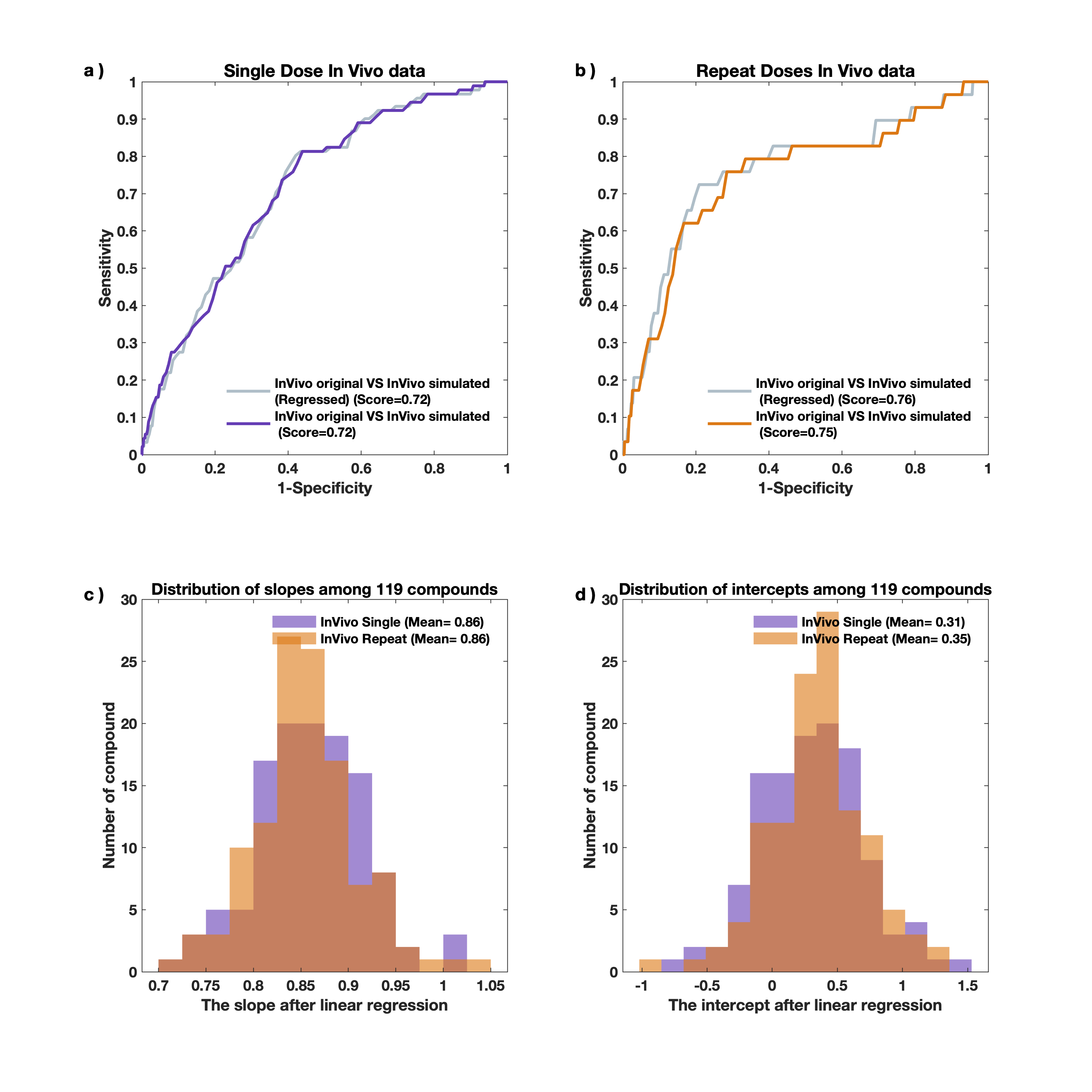
**

*Figure S4.* *The attributions of applying linear regression to in vitro and “drug-responding compound” in vivo data. Purple color and yellow color in this figure always indicate the data from single-dose and repeat-doses systems, respectively. a) and b) The consistencies between original in vivo data and simulated in vivo data obtained by PRank score. The gray lines indicate the scores obtained based on* *the simulated data (which are derived from applied linear regression between* *“drug-responding compound” and in vitro data) compared with original in vivo data. c) and d) The slope and intercept distributions, respectively, after launching linear regression between original in vivo and simulated in vivo data.*


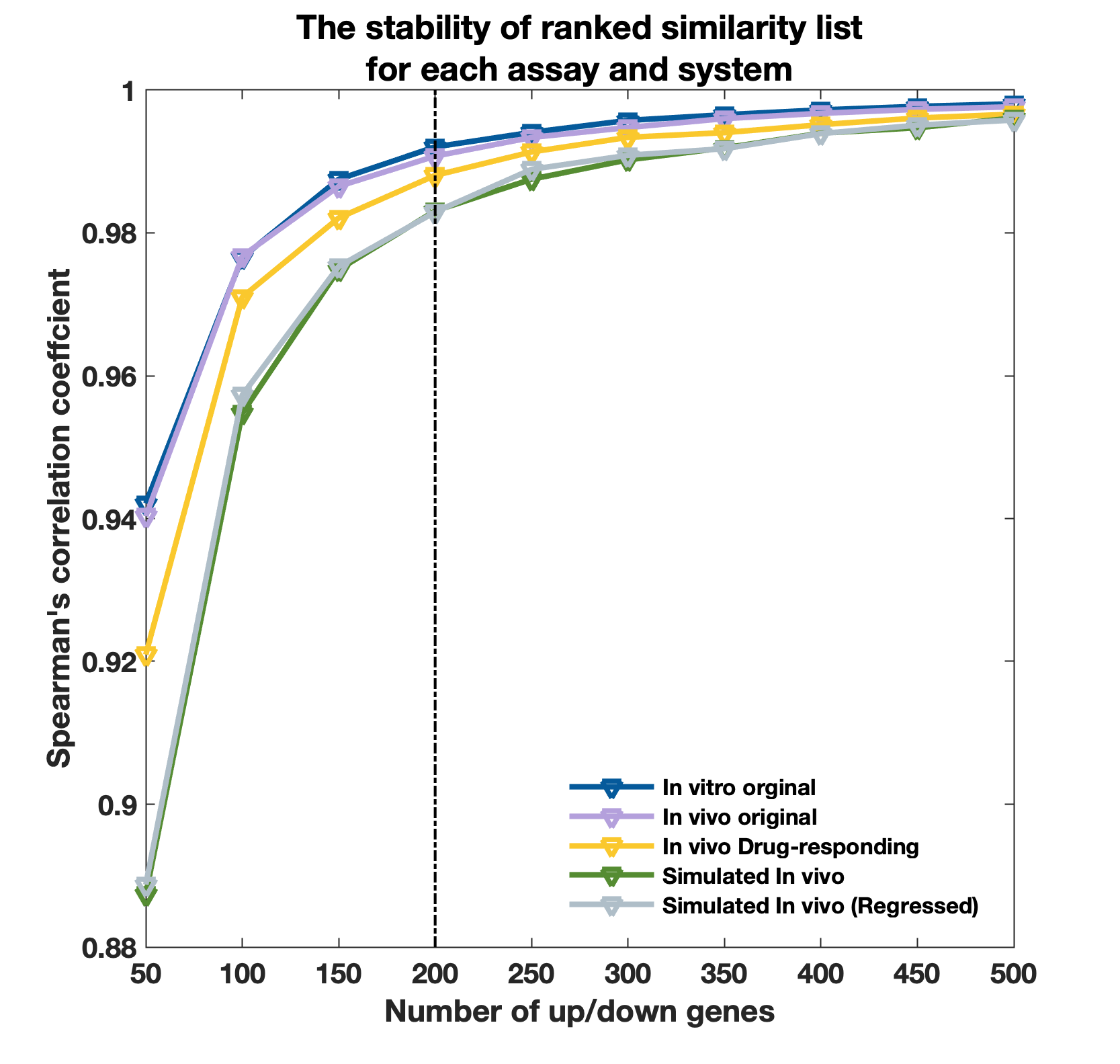


*Figure S5.* *The stability of the ranked similarity list for each assay and system. The data points were calculated by comparing the ranked similarity list with different numbers of differentially expressed genes by using Spearman’s correlation coefficient.*

*Table S1.* *The list of 119 common compounds*

| **No. (#)** | **Abbreviation** | **Compound** | **No. (#)** | **Abbreviation** | **Compound** |
| --- | --- | --- | --- | --- | --- |
| **1** | AA | allyl alcohol | **61** | GMC | gentamicin |
| **2** | AAF | acetamidofluorene | **62** | HCB | hexachlorobenzene |
| **3** | ACA | acarbose | **63** | HPL | haloperidol |
| **4** | ACZ | acetazolamide | **64** | HYZ | hydroxyzine |
| **5** | AJM | ajmaline | **65** | IBU | ibuprofen |
| **6** | AM | amiodarone | **66** | IMI | imipramine |
| **7** | AMT | amitriptyline | **67** | INAH | isoniazid |
| **8** | ANIT | naphthyl isothiocyanate | **68** | IPA | iproniazid |
| **9** | APAP | acetaminophen | **69** | KC | ketoconazole |
| **10** | APL | allopurinol | **70** | LBT | labetalol |
| **11** | ASA | aspirin | **71** | LS | lomustine |
| **12** | AZP | azathioprine | **72** | MDP | methyldopa |
| **13** | BBZ | bromobenzene | **73** | MEF | mefenamic acid |
| **14** | BBr | benzbromarone | **74** | MEX | mexiletine |
| **15** | BCT | bucetin | **75** | MFM | metformin |
| **16** | BDZ | bendazac | **76** | MP | methapyrilene |
| **17** | BEA | bromoethylamine | **77** | MTS | methyltestosterone |
| **18** | BZD | benziodarone | **78** | MTZ | methimazole |
| **19** | CAF | caffeine | **79** | MXS | moxisylyte |
| **20** | CAP | captopril | **80** | NFT | nitrofurantoin |
| **21** | CBP | carboplatin | **81** | NFZ | nitrofurazone |
| **22** | CBZ | carbamazepine | **82** | NIC | nicotinic acid |
| **23** | CFB | clofibrate | **83** | NIF | nifedipine |
| **24** | CHL | chlorpheniramine | **84** | NIM | nimesulide |
| **25** | CIM | cimetidine | **85** | NPAA | phenylanthranilic acid |
| **26** | CLM | chlormadinone | **86** | OPZ | omeprazole |
| **27** | CLT | cephalothin | **87** | PAP | papaverine |
| **28** | CMA | coumarin | **88** | PB | phenobarbital |
| **29** | CMN | chlormezanone | **89** | PCT | phenacetin |
| **30** | CMP | chloramphenicol | **90** | PEN | penicillamine |
| **31** | COL | colchicine | **91** | PH | perhexiline |
| **32** | CPA | cyclophosphamide | **92** | PHE | phenytoin |
| **33** | CPM | clomipramine | **93** | PML | pemoline |
| **34** | CPP | chlorpropamide | **94** | PMZ | promethazine |
| **35** | CPX | ciprofloxacin | **95** | PTU | propylthiouracil |
| **36** | CSA | cyclosporine A | **96** | PhB | phenylbutazone |
| **37** | CSP | cisplatin | **97** | QND | quinidine |
| **38** | DFNa | diclofenac | **98** | RAN | ranitidine |
| **39** | DIL | diltiazem | **99** | RIF | rifampicin |
| **40** | DIS | disopyramide | **100** | SLP | sulpiride |
| **41** | DNZ | danazol | **101** | SS | sulfasalazine |
| **42** | DOX | doxorubicin | **102** | SST | simvastatin |
| **43** | DSF | disulfiram | **103** | SUL | sulindac |
| **44** | DTL | dantrolene | **104** | TAA | thioacetamide |
| **45** | DZP | diazepam | **105** | TAC | tacrine |
| **46** | EBU | ethambutol | **106** | TAN | tannic acid |
| **47** | EE | ethinylestradiol | **107** | TBF | terbinafine |
| **48** | EME | erythromycin ethylsuccinate | **108** | TC | tetracycline |
| **49** | ENA | enalapril | **109** | TCP | ticlopidine |
| **50** | ET | ethionine | **110** | TEO | theophylline |
| **51** | ETN | ethanol | **111** | TIO | tiopronin |
| **52** | ETP | etoposide | **112** | TLB | tolbutamide |
| **53** | FAM | famotidine | **113** | TMD | trimethadione |
| **54** | FFB | fenofibrate | **114** | TMX | tamoxifen |
| **55** | FP | fluphenazine | **115** | TRI | triamterene |
| **56** | FT | flutamide | **116** | TZM | triazolam |
| **57** | FUR | furosemide | **117** | VA | vitamin A |
| **58** | GBC | glibenclamide | **118** | VPA | valproic acid |
| **59** | GF | griseofulvin | **119** | WY | WY-14643 |
| **60** | GFZ | gemfibrozil |  |  |  |

*Table S2. The sample information of validation dataset (NA: no information available, h: hour, d: day)*

|  | Available samples of *in vitro data set* | | | | | | Available samples of *in vivo* data set | | | | | | |
| --- | --- | --- | --- | --- | --- | --- | --- | --- | --- | --- | --- | --- | --- |
| Abbreviation | **Compound** | **Selection** | | **Dosage** | | | **Treatment** | | | | | | |
|  |  |  |  | **Low** | **Middle** | **High** | **6h** | **12h** | **48h** | **1d** | **3d** | **7d** | **14d** |
| 2-NF | 2-Nitrofluorene | | × | 3 | 3 | 3 | 0 | 0 | 0 | 0 | 3 | 3 | 0 |
| 2AAF | 2-Acetylaminofluorene | | √ | 3 | 3 | 3 | 0 | 0 | 0 | 3 | 3 | 3 | 3 |
| 3-MC | 3-Methylcholanthrene | | × | 3 | 3 | 2 | 0 | 0 | 0 | 3 | 3 | 3 | 3 |
| AA | Acetamide | | √ | 3 | 3 | 3 | 0 | 0 | 0 | 3 | 3 | 3 | 3 |
| Aap | Acetaminophen | | × | 3 | 3 | 3 | 3 | 3 | 2 | 4 | 0 | 0 | 0 |
| AfB1 | Aflatoxin B1 | | × | 3 | 3 | 3 | 0 | 0 | 0 | 0 | 3 | 3 | 0 |
| AlAl | Allyl alcohol | | √ | 3 | 3 | 3 | 0 | 0 | 0 | 3 | 3 | 3 | 3 |
| CFX | Cefuroxime | | √ | 3 | 3 | 3 | 0 | 0 | 0 | 3 | 3 | 3 | 3 |
| CIDB | C.I Direct Black | | × | 3 | 3 | 3 | 0 | 0 | 0 | 0 | 3 | 3 | 0 |
| Clon | Clonidine | | √ | 3 | 3 | 3 | 0 | 0 | 0 | 3 | 3 | 3 | 3 |
| CPA | Cyproterone acetate | | √ | 3 | 3 | 3 | 0 | 0 | 0 | 3 | 3 | 3 | 3 |
| DCB | 1,4-Dichlorobenzene | | √ | 3 | 3 | 3 | 0 | 0 | 0 | 3 | 3 | 3 | 3 |
| DEHA | Dehydroepiandrosterone | | × | 3 | 3 | 0 | 0 | 0 | 0 | 3 | 3 | 3 | 3 |
| DES | Diethylstilbestrol | | × | 3 | 3 | 3 | 0 | 0 | 0 | 2 | 3 | 0 | 0 |
| DMN | Dimethylnitrosamine | | × | 3 | 3 | 3 | 0 | 0 | 0 | 0 | 3 | 3 | 0 |
| ETH | Ethionine | | √ | 3 | 3 | 3 | 0 | 0 | 0 | 3 | 3 | 3 | 3 |
| Ibup | Ibuprofen | | √ | 3 | 3 | 3 | 0 | 0 | 0 | 3 | 3 | 3 | 3 |
| Mcarb | Methylcarbamate | | √ | 3 | 3 | 3 | 0 | 0 | 0 | 3 | 3 | 3 | 3 |
| MDA | Methylendianiline | | √ | 3 | 3 | 3 | 0 | 0 | 0 | 3 | 3 | 3 | 3 |
| MPy | Methapyrilene HCl | | × | 3 | 3 | 3 | 0 | 0 | 0 | 0 | 3 | 3 | 0 |
| Nif | Nifedipine | | √ | 3 | 3 | 3 | 0 | 0 | 0 | 3 | 3 | 3 | 3 |
| NNK | 4-(Methylnitrosamino)-1-(3-pyridyl)-1-butanone | | √ | 3 | 3 | 3 | 0 | 0 | 0 | 0 | 0 | 3 | 3 |
| NNM | N-Nitrososmorpholine | | × | 3 | 3 | 3 | 0 | 0 | 0 | 0 | 3 | 3 | 0 |
| NPip | N-Nitrosopiperidine | | √ | 3 | 3 | 3 | 0 | 0 | 0 | 3 | 3 | 3 | 3 |
| PB | Phenobarbital | | NA | NA | NA | NA | 0 | 0 | 0 | 3 | 3 | 3 | 3 |
| PBO | Piperonylbutoxide | | × | 3 | 3 | 3 | 0 | 0 | 0 | 3 | 3 | 0 | 0 |
| Praz | Prazosin | | × | 3 | 3 | 3 | 0 | 0 | 0 | 3 | 3 | 3 | 3 |
| Prop | Propranolol | | √ | 3 | 3 | 3 | 0 | 0 | 0 | 3 | 3 | 3 | 3 |
| TAA | Thioacetamid | | × | 3 | 3 | 3 | 0 | 0 | 0 | 0 | 3 | 3 | 0 |
| Wy | Wy-14643 | | × | 3 | 3 | 3 | 0 | 0 | 0 | 3 | 3 | 0 | 0 |

*Table S3. The* $W_{env}^{*}$ *and the average of* $H_{env}^{*}$ *obtained by* *single-dose in vivo data (format as .xlsx)*

[*OUTPUT_S.xlsx*](OUTPUT_S.xlsx)

*Table S4. The* $W_{env}^{*}$ *and the average of* $H_{env}^{*}$ *obtained by repeat-doses in vivo data (format as .xlsx)*

*<OUTPUT_R.xlsx>*

*Table S5.* *The* $W_{env}^{*}$ *and the average of* $H_{env}^{*}$ *obtained by validation in vivo data (format as .xlsx)*

[*OUTPUT_V.xlsx*](table_S5_OUTPUT_V.xlsx)
